# Overt speech critically changes lateralization index and did not allow determination of hemispheric dominance for language: an fMRI study

**DOI:** 10.1186/s12868-021-00671-y

**Published:** 2021-12-01

**Authors:** David Hassanein Berro, Jean-Michel Lemée, Louis-Marie Leiber, Evelyne Emery, Philippe Menei, Aram Ter Minassian

**Affiliations:** 1 Department of Neurosurgery, University Hospital of Caen Normandy, Avenue de la Côte de Nacre, 14000 Caen, France; 2 Normandie Univ, UNICAEN, CEA, CNRS, ISTCT/CERVOxy group, GIP Cyceron, Caen, France; 3 INSERM, CRCINA, Team 17, IRIS building, Angers, France; 4 Department of Neurosurgery, University Hospital of Angers, Angers, France; 5 Department of Radiology, University Hospital of Angers, Angers, France; 6 INSERM, UMR-S U1237, PhIND group, GIP Cyceron, Caen, France; 7 Department of Anesthesiology, University Hospital of Angers, Angers, France; 8 LARIS, ISISV team, University of Angers, Angers, France

**Keywords:** fMRI, Overt, Functional laterality, Language network, Cingulo-opercular network, Ventral attention network, Resting-state

## Abstract

**Background:**

Pre-surgical mapping of language using functional MRI aimed principally to determine the dominant hemisphere. This mapping is currently performed using covert linguistic task in way to avoid motion artefacts potentially biasing the results. However, overt task is closer to natural speaking, allows a control on the performance of the task, and may be easier to perform for stressed patients and children. However, overt task, by activating phonological areas on both hemispheres and areas involved in pitch prosody control in the non-dominant hemisphere, is expected to modify the determination of the dominant hemisphere by the calculation of the lateralization index (LI).

**Objective:**

Here, we analyzed the modifications in the LI and the interactions between cognitive networks during covert and overt speech task.

**Methods:**

Thirty-three volunteers participated in this study, all but four were right-handed. They performed three functional sessions consisting of (1) covert and (2) overt generation of a short sentence semantically linked with an audibly presented word, from which we estimated the “Covert” and “Overt” contrasts, and a (3) resting-state session. The resting-state session was submitted to spatial independent component analysis to identify language network at rest (LANG), cingulo-opercular network (CO), and ventral attention network (VAN). The LI was calculated using the bootstrapping method.

**Results:**

The LI of the LANG was the most left-lateralized (0.66 ± 0.38). The LI shifted from a moderate leftward lateralization for the Covert contrast (0.32 ± 0.38) to a right lateralization for the Overt contrast (− 0.13 ± 0.30). The LI significantly differed from each other. This rightward shift was due to the recruitment of right hemispheric temporal areas together with the nodes of the CO.

**Conclusion:**

Analyzing the overt speech by fMRI allowed improvement in the physiological knowledge regarding the coordinated activity of the intrinsic connectivity networks. However, the rightward shift of the LI in this condition did not provide the basic information on the hemispheric language dominance. Overt linguistic task cannot be recommended for clinical purpose when determining hemispheric dominance for language.

**Supplementary Information:**

The online version contains supplementary material available at 10.1186/s12868-021-00671-y.

## Background

Most functional magnetic nuclear resonance imaging (fMRI) studies of language aiming to map cortical activity for presurgical planning are performed covertly [1], in fact, performing an overt linguistic task during fMRI potentially expose to motion artefacts, decreasing the reliability of language mapping. The alternative method to avoid motion artefacts while overtly performing a linguistic task consists in sparse acquisition. Initially described for the study of the audition to ensure that the stimulus was not masked by the scanner noise [2], it has been applied to study the overt speech. In such acquisition, one exploits the fact that the hemodynamic response function to neuronal activity is delayed. Thus, the acquisition is made during a brief period following an instruction to suspend speech. This method has been widely used for scientific purpose aiming to explore all aspects of language including phonology [3, 4]. However, even though this method is valid for scientific purpose in group studies, it is not suitable for presurgical mapping of language. Indeed, in this situation the clinician expects statistical mapping at the individual-level, and unfortunately, the sparse acquisition, due to the brief acquisition periods, has a poor signal-to-noise ratio and give poor statistical information at the individual level.

Recently, some authors preconize to perform overtly linguistic task especially in pediatric patient for which overt task may be easier to perform because of the more “ecological” conditions [5–11]. We have shown that overt speaking in modern fMRI scanners induced no identifiable artifacts and gave robust statistical results at the individual-level [12], seemingly circumventing the problem of the poor signal-to-noise ratio inherent to sparse acquisition.

In clinical studies of speech, one should keep in mind that for most neurosurgeons, the presurgical mapping of language aimed to determine the hemispheric dominance of language as published in this recent interesting survey (this was the goal of 92% of ordered fMRI) [13]. This is generally assessed by the calculation of the lateralization index (LI) of the statistical map obtained from the task-induced brain activity [14]. Clinical evidences and neuroimaging studies have shown that the language is implemented along the left sylvian fissure [15, 16]. However, despite its left lateralization, the literature shows that the lateralization of the language network is dependent on the syntactic or semantic nature of the task being executed as well as the difficulty and amount of the required semantic integration. It is well known that semantic ambiguities of specific linguistic tasks including poetics, metaphors comprehension, and the focus on the literal signification of idiomatic expression, are recruiting non-dominant hemisphere [17–37].

Furthermore, the right hemisphere contributes to some important aspect of language such as pitch prosody comprehension and production [38–40], supported by the right hemispheric white matter bundles connecting the right lateralized ventral attention network (VAN) [41].

In our recent study, we have shown that overt speaking in fMRI, compared to covert session, using a simple word sentence matching task, with no or minimal content in semantic ambiguities and prosodic intonation, allowed identification of the areas involved in the sensory feedback of prosody in the right hemisphere (increased activity in the right superior temporal sulcus and gyrus, extending to the Heschl gyri), together with the areas involved in the bilateral primary motor control of speech (increased activity in bilateral pre- and post-central gyri and cerebellar cortex). In addition, we observed that the areas within the cingulo-opercular network (CO) [12] were automatically activated by the saliency of acoustic inputs.

Furthermore, we showed in this study that motion artefacts did not significantly affect fMRI data analyses. Indeed, we did not find the typical artifacts described in insular and deep opercular areas [8] when we compare the overt to the covert session, with or without regression of the movement parameters [12]. Overall, even though movement parameters significantly differed in the y and z directions when comparing overt to covert tasks, they did not exceed 3 mm in translation and 2° in rotation during both tasks. These movement artefacts did not affect the results as indicated by the similarity of the images obtained by neglecting movement parameters or considering them as nuisance covariates. Additional file 1: Table S1 shows movement parameters for all subjects in the overt and covert sessions.

All the described activations when overtly performing a linguistic task, are expected to more or less modify the lateralization of the language mapping. Because, as previously mentioned, a major information for neurosurgeons concerns the hemispheric dominance for language [42–44], a careful analysis of the lateralization induced by the overt speech is a prerequisite before proposing its use for presurgical mapping.

The data set of the present work is the same as in our precedent paper dealing with the feasibility of overt speech in fMRI [12]. Here we analyzed the LI of the brain activity obtained from the covert and overt linguistic tasks we routinely used for language presurgical mapping in patients with brain gliomas. Furthermore, in this study, we took advantage from the realization by the same subjects of a resting-state session to perform a connectivity analysis, and that to isolate three intrinsic connectivity networks (ICN): CO, VAN and language network (LANG) [45, 46]. Indeed, recent clinical studies suggest that the connectivity analysis at rest allows identification of the areas involved in language with great sensitivity. This complementary connectivity analysis may allow us to better understand the dynamic cooperation of ICNs and their influence on the lateralization of the brain activity. Here we show that, the overt linguistic task results in a critical shift of the speech-induced cortical activity towards the right hemisphere, thus, challenging the use of the overt linguistic task for presurgical mapping of language.

## Methods

They were identical to those of our previous work [12] but differed by a connectivity analysis and focused on the LI.

### Participants

Thirty-three volunteers (17 male subjects) participated in the study (mean age was 25.9 ± 5.3 years). The subjects were all native French speakers with no neurologic diseases. This study was approved by the Local Ethics Committee (Comité de protection des personnes, CPP Ouest II, Angers, France, authorization date: November 15, 2012). All subjects gave their written, informed consent prior to their enrollment in this study. All methods were carried out in accordance with the relevant guidelines and regulations.

### Experimental design

The tasks in two of the functional sessions consisted of the covert or overt generation of a short sentence semantically linked with an audibly presented word through the acoustic headset of the MRI.

Before scanning and after completing the Edinburgh handedness inventory (i.e., Edinburgh inventory score; EIS) [47], the subjects were instructed that the overt speaking was not done in way to be heard but to activate phonologic areas. They were briefly trained to avoid excessive head and jaw movements in the task. A different word was presented every 4 s four times. This 16-s block of “sentence generation” alternated with a 16-s “tones-listening” block of 4-s tones of three increasing frequency covering approximately the audible band, presented four times (an illustrative example can be found in Additional file 1: Fig. S1, as published in our previous paper [12]).

A resting-state session and a continuous linguistic task session of equal duration were also performed for functional connectivity analysis. The continuous linguistic task is not treated in the present study. The order of sessions was randomly assigned across subjects.

### MRI protocol

All structural and functional MRI data were acquired on a Siemens 3 T MRI (Magnetom Skyra, Siemens Medical Systems, Erlangen, Germany). The structural images were acquired using a 3D T1-weighted imaging (3DT1w) protocol with the following scan parameters: TE = 3.21 ms, TR = 2000 ms, matrix = 256 × 256, FOV = 255 × 284 mm, FA = 8°, and voxel size 1 × 1 × 1 mm. Functional images were collected using gradient-echo EPI sequence with the following parameters: TR = 2280 ms, TE = 30 ms, FA = 90°, FOV = 168 × 187 mm, 40 axial slices and voxel size = 3 × 3 × 4 mm. A total of 270 cerebral volumes were acquired in each session. Four functional sessions were performed for every subject.

### fMRI preprocessing

Data analysis was performed using Statistical Parametric Imaging SPM12 (the Wellcome Department of Imaging, Institute of Neurology, University College London) running on MATLAB (The MathWorks, Natick, MA). The Computational Anatomy Toolbox CAT12 was used for segmentation and normalization, and the Anatomy toolbox for SPM12 was used for the detection of clusters peaks and anatomical classification.

After slice timing, functional images were first realigned across trials and then coregistered to the structural image using the segmented gray matter as the reference image and the mean functional image as the source. The realigned functional images were spatially normalized to the template in Montreal Neurological Institute (MNI) space and then smoothed using an isotropic Gaussian kernel of 6 mm full width at half maximum (FWHM). For all functional sessions, we used unwarping in the image processing to correct movement-related geometric distortions in regions where there is an air-tissue interface (susceptibility-by-movement interaction).

### Data and statistical analysis

#### First-level analysis

Parametric maps were computed within the gray matter masks. Statistical significance was fixed to *p* = 0.05 family-wise error (FWE) corrected for multiple comparisons at the voxel-level and a minimum cluster extent of 5 pixels. We performed two main contrasts: covert > tones (“Covert”, − 1, 1) and overt > tones (“Overt”, − 1, 1), both for the isolation of language-activated brain areas. Note that we used capital letters to specifically designate the name of a contrast (i.e., Covert or Overt). Statistics for these two contrasts were performed with movement parameters regression as confounding variables.

The resting-state session was submitted to spatial independent component analysis (sICA) using a customized version of the Infomax algorithm [45] running under MATLAB, for the identification of large‐scale networks [48] and calling 55 components. Statistical significance was fixed at Z = 2 standard deviation at the individual level. One of the main difficulties of this method is the determination of the total number of components to be used, which may lead to suboptimal decompositions with the merging of multiple networks in case of a low number, or the fragmentation of a functional network into multiple components in case of a high number [49, 50]. Our choice to analyze 55 components among all patients was based on a previous work and appeared to be a good compromise [51]. We did not use automatic identification of the functional networks of interest, but we used traditional visual inspection. Even though this method is time‐consuming and sometimes biased, our experience showed that it allows identification of language network with a high reliability [12, 41, 45, 46].

Statistics for the resting-state session were performed with regression, as nuisance covariates, of the movement parameters and of the signal extracted from the white matter and the LCS exclusion mask without global signal regression.

ICN’s identification was performed by two experts (ATM, JML). The LANG was identified at the individual level using the same criteria as in our previous studies with a special emphasis for the posterior superior temporal sulcus (pSTS) activity [45, 46]. The CO was identified as a network showing activity in the dACC, AIFO and anterior middle frontal gyrus. The VAN was identified as an ICN showing strong similarities with the language network but generally right-lateralized and showing activity in the inferior parietal lobule (IPL) within the temporo-parietal junction (TPJ) in the vicinity of supramarginal gyrus (SMG) and thus more anterior to the IPL activity of the LANG within the angular gyrus (ANG) [45].

Note that, in this study we choose to not perform sICA on the overt and covert sessions. As we showed that covert task activated components of the CO network and overt task activated components of both CO and VAN networks [12], sICA on block-designed sessions can potentially aggregates these networks.

#### Second-level analysis

A univariate Student’s t-test was performed as second-level group analysis for the contrasts Covert and Overt and for the LANG, CO and VAN identified at rest. The statistical maps were FWE corrected for multiple comparisons at the voxel-level with a minimum extent of k = 5 pixels.

Complementary analysis included one sample t-tests of the Covert and Overt contrasts within the masks FWE corrected 0.05 at the cluster-level of the LANG, CO, and VAN.

When corrected at the cluster-level, *p* was fixed to 0.001 at the voxel-level.

The LI [52] was calculated on unthresholded first-level analysis t-maps of the Overt and Covert contrasts using the bootstrapping method [53] with the parameters of intensity threshold = 0 (positive t-maps), sample size = 25%, maximal and minimal number of voxels = 100,000 and 5, respectively.

In general, the LI value is computed using the following formula:

$$LI = f.\frac{{Q_{LH} - Q_{RH} }}{{Q_{LH} + Q_{RH} }}$$

where Q_LH_ and Q_RH_ are representative quantities measured by fMRI for the left (LH) and right (RH) hemisphere contributions, respectively. The factor f is a scaling factor that defines the range of LI values (i.e., LI varies continuously from − f for pure RH dominance to + f for pure LH dominance) [52].

In way to track bias in the calculation and to ensure that the possible shift of the LI was not specific to the lobar anatomy, we used special masks, in addition to the whole brain mask:

- To reduce the bias induced by the cross cerebellar activity, the LI was calculated on t-maps of both contrasts (Covert and Overt) and of the three networks (LANG, VAN, and CO) using the cerebellum as exclusive mask.
- We ensured that the LI may not differ between Overt and Covert contrasts in specific areas. For this purpose, the LI for these two contrasts was calculated in the specific masks of the temporal (LIt), parietal (LIp) and frontal areas (LIf) provided by the LI-Toolbox for SPM12.
- As in this study we were interested in nontrivial modifications of the LI, such those induced by the speech motor activity that was expected to be bilateral and greater in the Overt condition, the analysis was also performed using pre and postcentral gyrus as exclusive masks for frontal and parietal areas respectively, and that for the Overt and Covert contrasts. These binary masks were produced using the AAL template provided by the MRIcron software.
- Whole brain and sus-tentorial LI were also calculated for both contrasts within the cluster-level corrected mask of the CO. This was done to track changes of the activity lateralization within the CO between the Overt and Covert conditions.

Complementary statistical analyses included t-tests, linear regression, and analysis of variance with Tukey’s multiple comparison test as required (GraphPad Prism®).

## Results

Thirty-three volunteers (17 males) were enrolled in this study, and four were left-handed according to the EIS. All volunteers completed the three sessions, i.e., the overt and covert sentence generation sessions and the resting-state session.

**Fig. 1:**
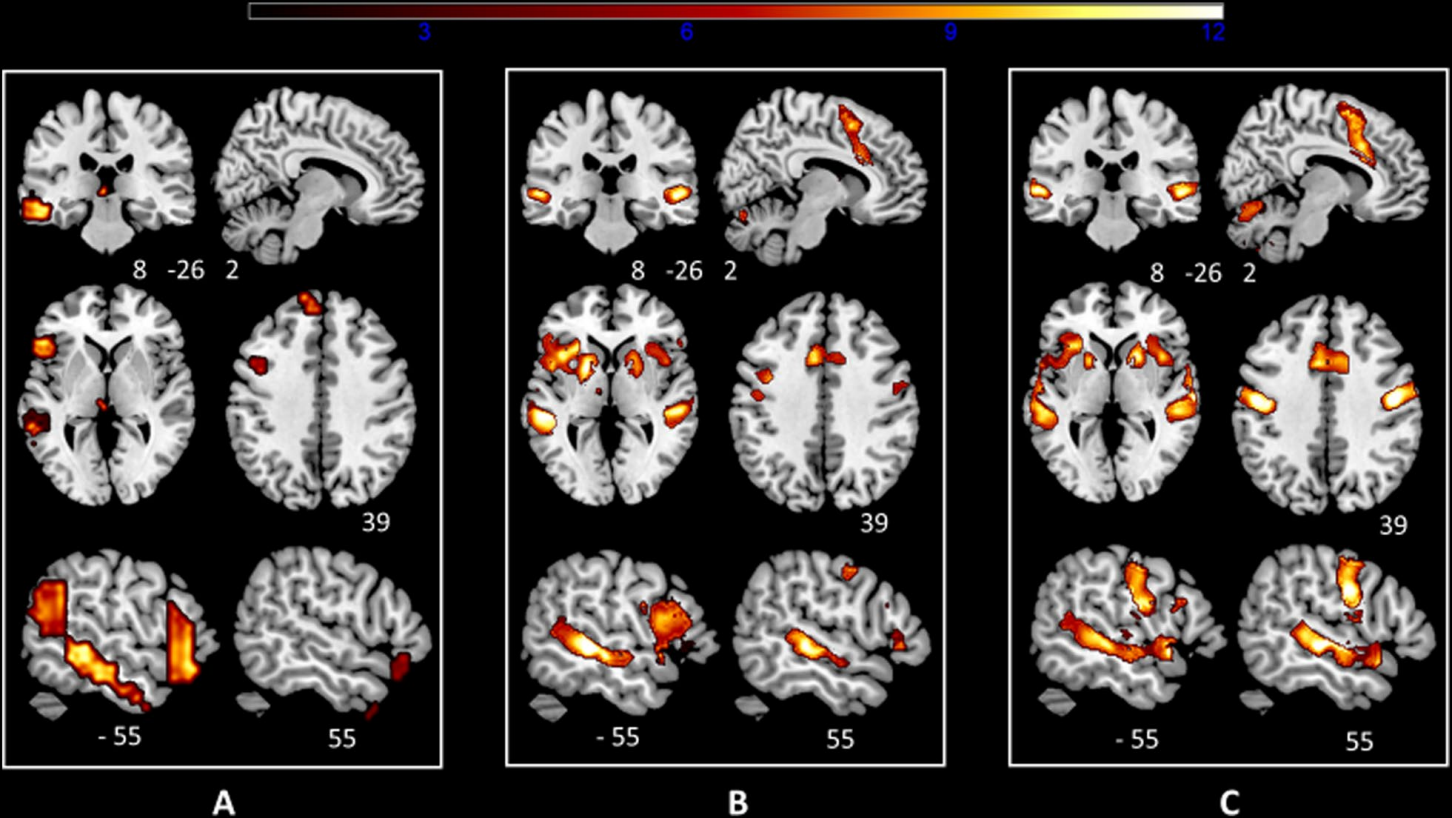
One-sample t-test group analysis. **A** The language network as identified by functional connectivity analysis at rest (LANG). **B** The contrast covert speech production > tones-listening (Covert). **C** The contrast overt speech production > tones-listening (Overt). Images are projected on an MNI template. The left side of the brain is on the left. Images are shown corrected for multiple comparison FWE = 0.05 at the voxel-level with a minimum cluster extent k = 5. The color bar indicates t-values. White numbers are the coordinates of the corresponding slices. Note the increasing recruitment of the right middle temporal gyrus/superior temporal sulcus from (**A**) to (**C**) together with the laryngeal motor area within the precentral gyrus. Note also in **B** and **C** the strong activity induced by the explicit task within the main nodes of the cingulo-opercular network (AIFO, dACC, putamen) and the rightward shift in AIFO and putamen activity during the overt task

The Covert and Overt contrasts showed robust leftward shifted activity in the inferior frontal, middle temporal, and angular gyri and crossed the cerebellar activity. The Overt contrast showed supplementary bilateral motor activity from lips and mouth areas and larynx phonologic areas as well as bilateral motor cerebellar activity and increased right middle temporal gyrus activity (Fig. 1B and C).

**Fig. 2:**
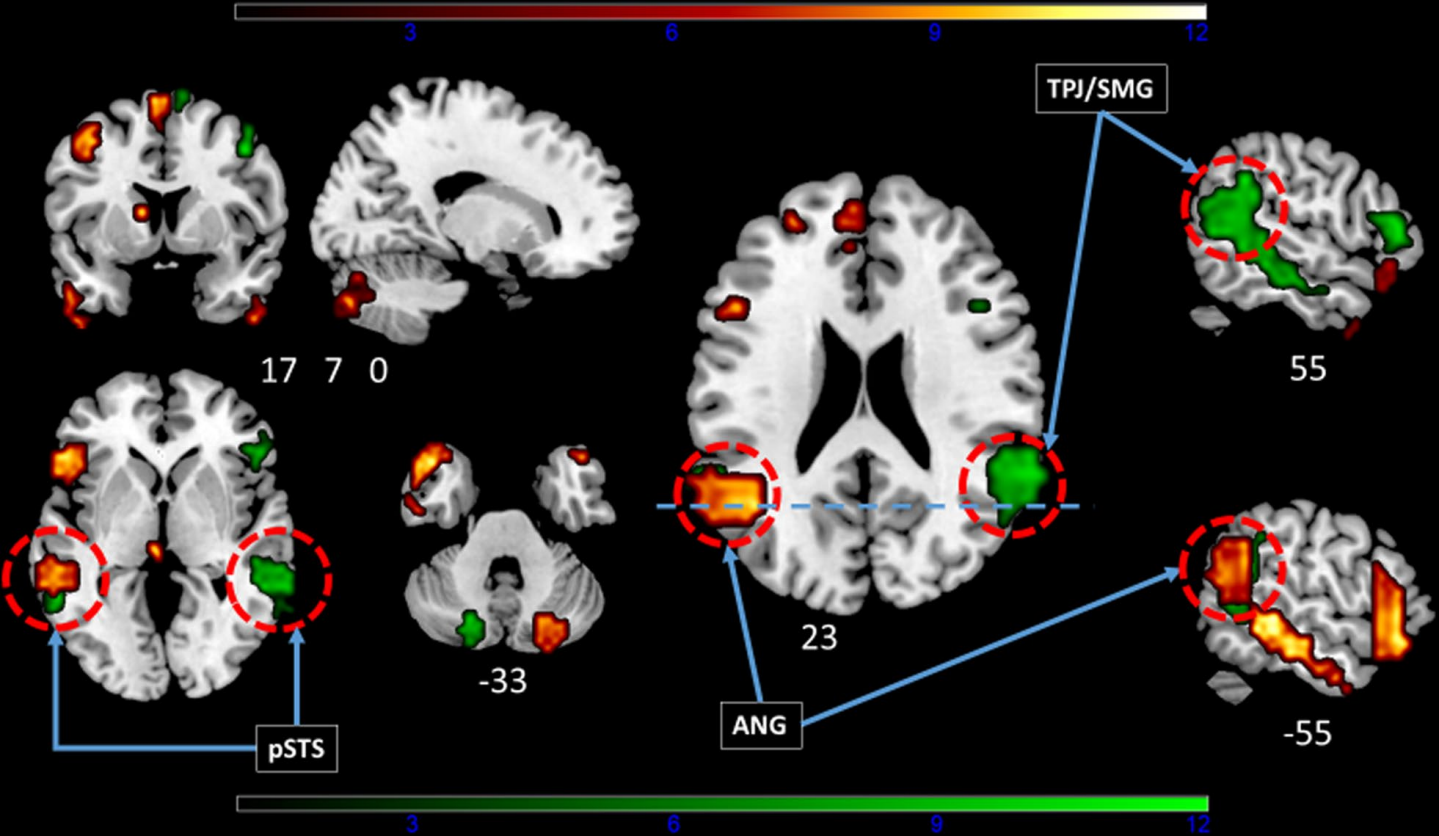
One-sample t-test group analysis. The language network (LANG, n = 32) shown in hot and the ventral attentional network (VAN, n = 33) in green as identified by functional connectivity analysis at the group level projected on an MNI template. The left side of the brain is on the left. Images are shown corrected for multiple comparison FWE = 0.05 at the voxel-level with a minimum cluster extent k = 5. Color bars indicate t-values. White numbers are the coordinates of the corresponding slices. ANG: angular gyrus, TPJ: temporo-parietal junction, SMG: supramarginal gyrus, pSTS: posterior superior temporal sulcus. Note the symmetric pattern of activity in these two intrinsic connectivity networks, including the pSTS. These two networks can be discriminated in atypically lateralized subjects by the peak of activity within the inferior parietal lobule involving the ANG for the LANG and the more anterior TPJ for the VAN

**Fig. 3:**
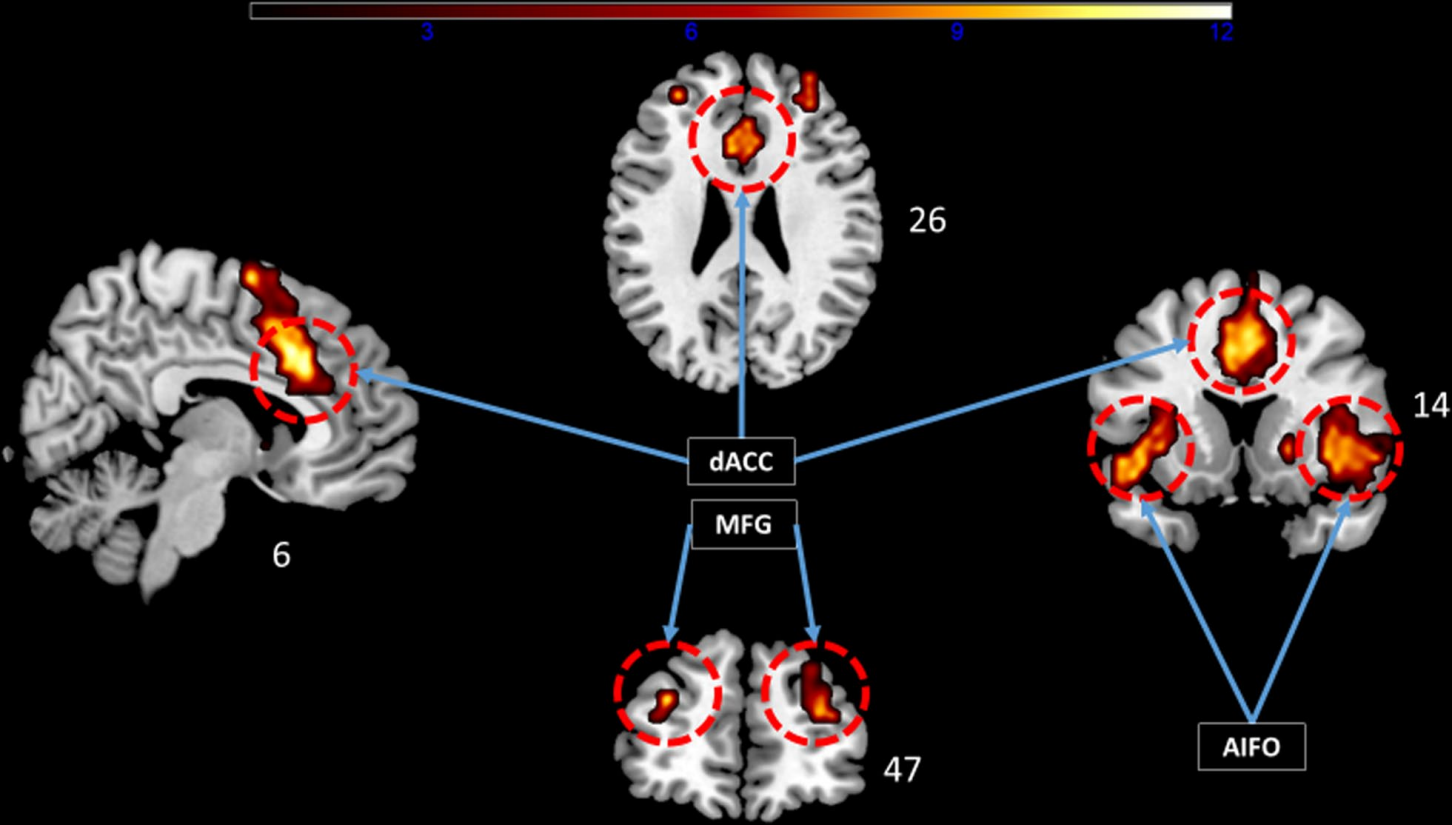
One-sample t-test group analysis. The cingulo-opercular network (CO, n = 33) shown in hot as identified by functional connectivity analysis at the group-level projected on an MNI template. The left side of the brain is on the left. Images are shown corrected for multiple comparison FWE = 0.05 at the voxel-level with a minimum cluster extent k = 5. The color bar indicates t-values. White numbers are the coordinates of the corresponding slices. dACC: dorsal anterior cingulate, AIFO: anterior insula-frontal operculum, MFG: middle frontal gyrus. Note the right lateralization of activity within the AIFO

Intrinsic connectivity analysis by sICA at rest allowed identification of the LANG and the CO in 32 subjects and the VAN in all subjects (Figs. 2 and 3, Tables 1, 2, 3). Descriptive statistics of the LI values for each contrast (Overt and Covert contrasts), and for each resting-state ICN (LANG, CO, and VAN), with the different used masks, are represented in Table 4.

**Table 1:** One-sample t-test, group-level, functional connectivity analysis (n = 33). Peaks of components of the language network at rest as identified by spatial independent component analysis

	**k**	**t**_**32**_	**x**	**y**	**z**
L IFG (p. Orbitalis)	850	12.88	− 47	28	− 16
L Middle temporal gyrus		11.23	− 59	− 36	− 4
L Medial temporal pole		10.17	− 44	20	− 32
L Middle temporal gyrus		10.15	− 55	− 25	− 16
L Angular gyrus		9.94	− 44	− 55	23
L Middle frontal gyrus		9.55	− 40	8	52
L Inferior temporal gyrus		7.48	− 52	1	− 40
L Inferior temporal gyrus		6.55	− 52	− 10	− 32
L Middle frontal gyrus		6.41	− 47	20	36
L IFG (p. Opercularis)		6.39	− 44	16	32
L PreSMA	211	9.93	− 6	23	59
L PreSMA		9.14	− 6	12	67
L Superior frontal gyrus		7.76	− 13	57	36
L Superior medial gyrus		7.52	− 10	50	27
L Superior medial gyrus		7.46	− 6	49	39
L ACC		6.27	− 6	50	11
L Middle frontal gyrus		6.03	− 28	50	24
R Cerebellum (Crus 2)	124	9.27	23	− 82	− 41
R Cerebellum (Crus 1)		8.73	23	− 74	− 33
R Temporal pole	56	7.5	50	27	− 21
R Temporal pole		7.38	46	19	− 25
R Inferior temporal gyrus	22	7.01	50	8	− 45
L Caudate nucleus	17	7.92	− 10	5	11
L Thalamus	8	8.11	− 3	− 25	− 1
L Superior parietal lobule	5	6.01	− 33	− 63	47

*k* cluster extent in voxel number, *t*_*32*_ t-value for 32 degrees of freedom, *x, y, z* coordinates in MNI space, *ACC* anterior cingulate cortex, *IFG* inferior frontal gyrus, *SMA* supplementary motor area

**Table 2:** One-sample t-test, group-level, functional connectivity analysis (n = 33). Peaks of components of the ventral attentional network at rest as identified by spatial independent component analysis

	**k**	**t**_**32**_	**x**	**y**	**z**
R Temporo−parietal junction	368	10.45	64	− 45	22
R Middle temporal gyrus		8.09	57	− 37	− 2
R Middle temporal gyrus		7.3	49	− 30	− 10
R Middle temporal gyrus		6.76	49	− 7	− 22
L Middle temporal gyrus	85	7.45	− 59	− 52	6
L Temporo−parietal junction		7.37	− 59	− 45	22
R IFG (p. Triangularis)	60	8.36	53	31	6
R IFG (p. Triangularis)		7.51	49	27	− 2
R IFG (p. Triangularis)		6.16	42	19	22
L Cerebellum (Crus 2)	50	8.73	− 18	− 78	− 34
L Cerebellum (Crus 2)		8.72	− 18	− 78	− 42
R Middle frontal gyrus	30	7.56	46	1	54
R Precentral gyrus		6.62	46	8	46
L Precuneus	19	7.08	1	− 48	46
R PreSMA	15	7.59	12	12	70

*k* cluster extent in voxel number, *t*_*32*_ t-value for 32 degrees of freedom, *x, y, z* coordinates in MNI space, *IFG* inferior frontal gyrus, *SMA* supplementary motor area

**Table 3:** One-sample t-test, group-level, functional connectivity analysis (n = 33). Peaks of components of the cingulo-opercular network at rest as identified by spatial independent component analysis

	**k**	**t**_**32**_	**x**	**y**	**z**
R MCC/dACC	238	12.14	4	19	38
R PreSMA		6.96	4	4	66
R PreSMA		6.86	8	1	70
L PreSMA		6.09	− 7	1	58
R Insula lobe (AIFO)	101	9.24	38	16	− 6
R Temporal pole		6.3	57	12	− 2
L Insula lobe (AIFO)	98	8.35	− 44	12	− 10
L Insula lobe (AIFO)		7.57	− 33	16	6
L Temporal pole		6.9	− 52	8	− 6
R Middle frontal gyrus	34	6.81	31	46	26
R Middle frontal gyrus		6.2	34	57	22
R Superior frontal gyrus		6.05	27	46	38
L Middle frontal gyrus	9	6.41	− 29	46	26
R Putamen	8	7.18	19	16	− 2
R Caudate nucleus		5.89	*8*	8	− 2

*k* cluster extent in voxel number, *t*_*32*_ t-value for 32 degrees of freedom, *x, y, z* coordinates in MNI space, *ACC* anterior cingulate cortex, *AIFO* anterior insula frontal operculum, *SMA* supplementary motor area

**Table 4:** Descriptive statistics of the LI values for each contrast (Overt and Covert contrasts), and for each resting− state network (LANG, CO, and VAN), with the different used masks

Mask	Number of values	Minimum	Maximum	Range	Mean	Standard deviation
Covert						
Whole brain	33	− 0.65	0.76	1.41	0.2163	0.3208
Sus-tentorial	33	− 0.84	0.86	1.7	0.3292	0.3793
Frontal	33	− 0.79	0.88	1.67	0.4248	0.3645
Frontal, preCG excluded	33	− 0.76	0.84	1.6	0.4579	0.3374
Parietal	33	− 0.8	0.94	1.74	0.3688	0.4085
Parietal, postCG excluded	33	− 0.85	0.94	1.79	0.3243	0.443
Overt						
Whole brain	33	− 0.63	0.44	1.07	− 0.1855	0.275
Sus-tentorial	33	− 0.67	0.62	1.29	− 0.136	0.3043
Frontal	33	− 0.5	0.73	1.23	− 0.05997	0.3346
Frontal, preCG excluded	33	− 0.38	0.61	0.99	0.03855	0.288
Parietal	33	− 0.59	0.75	1.34	-0.02548	0.364
Parietal, postCG excluded	33	− 0.73	0.68	1.41	-0.1892	0.3413
LANG						
Whole brain	32	− 0.74	0.85	1.59	0.5931	0.3221
Sus-tentorial	32	− 0.77	0.91	1.68	0.6572	0.3402
CO						
Whole brain	32	− 0.69	0.69	1.38	− 0.1179	0.3718
Sus-tentorial	32	− 0.7	0.52	1.22	− 0.1564	0.3575
VAN						
Whole brain	33	− 0.86	0.41	1.27	− 0.4808	0.3925
Sus-tentorial	33	− 0.88	0.4	1.28	− 0.4991	0.403

*CO* cingulo−opercular network, *LANG* language network, *postCG* postcentral gyrus, *preCG* precentral gyrus, *VAN* ventral attention network

The LANG presented right hemispheric temporal components restricted to small clusters in the temporal poles and, indeed, no components of the CO. The Covert and the Overt contrasts both presented increased activity in the main nodes of the CO (AIFO and dACC), in addition to a right temporal activity which was greater in the Overt contrast (Fig. 1).

**Fig. 4:**
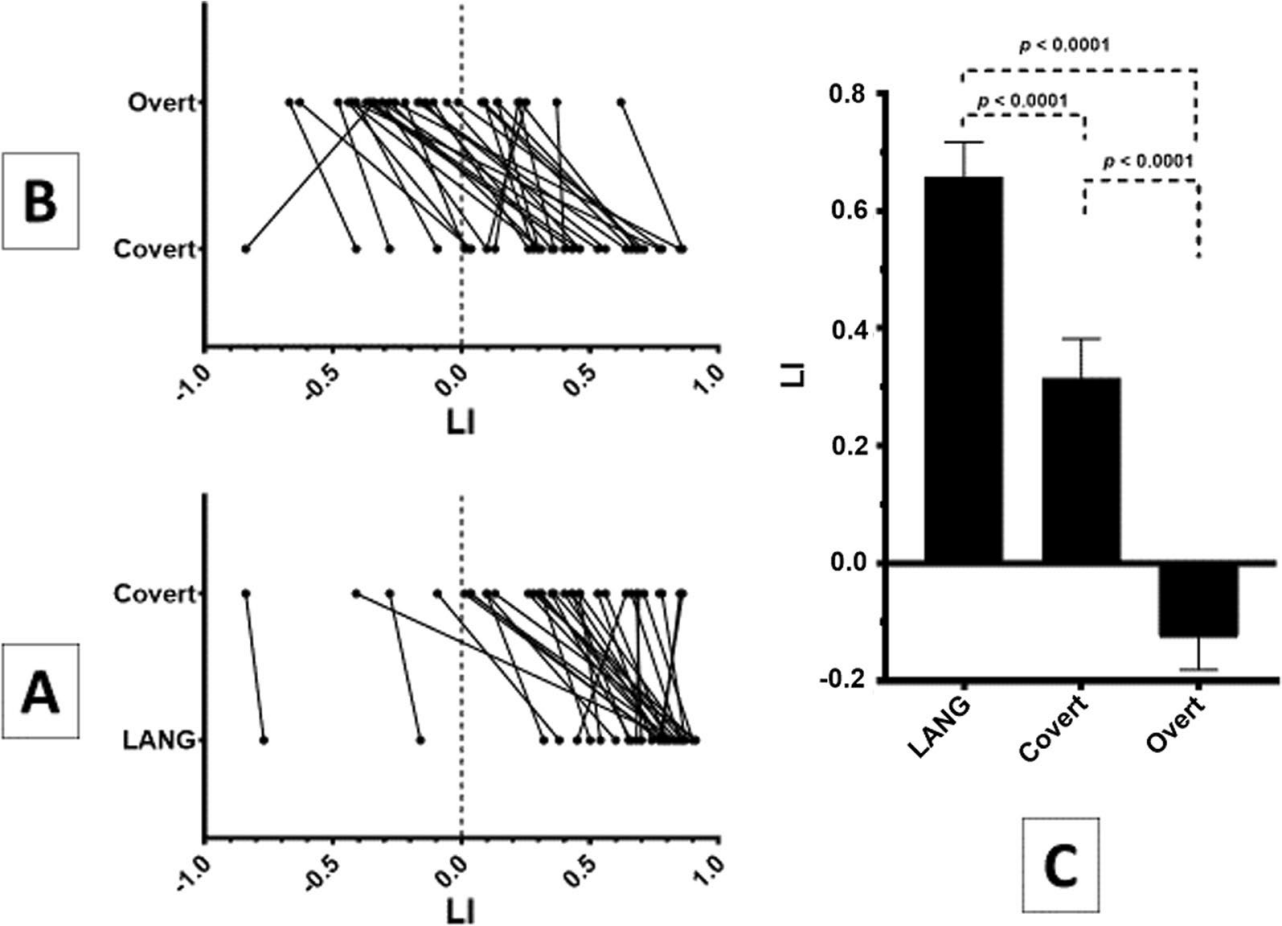
Sus-tentorial laterality index (LI) of the resting-state language network (LANG) identified by spatial independent components analysis and of the contrast sentence generation > tones-listening for the covert and overt sessions (Covert and Overt contrasts). Sus-tentorial LI for the explicit tasks were calculated using pre- and postcentral gyri as the exclusive mask. **A** The LI shift from the resting-state to the covert session (n = 32). **B** The LI shift from the covert to the overt session (n = 33). **C** One-way analysis of variance (n = 32). Positive LI indicates left lateralization

No correlation was found between the EIS and the LI in the Covert, Overt and LANG conditions. The LI of the LANG was the most left-lateralized (0.66 ± 0.38). The LI shifted from a moderate leftward lateralization for the Covert condition (0.32 ± 0.38) to a right lateralization in the Overt condition (− 0.13 ± 0.30). The LI of the LANG, Covert and Overt significantly differed from each other (Fig. 4). The same pattern of rightward shift was observed for the LI in the specific temporal, parietal and frontal masks (Additional file 1: Fig. S2).

**Fig. 5:**
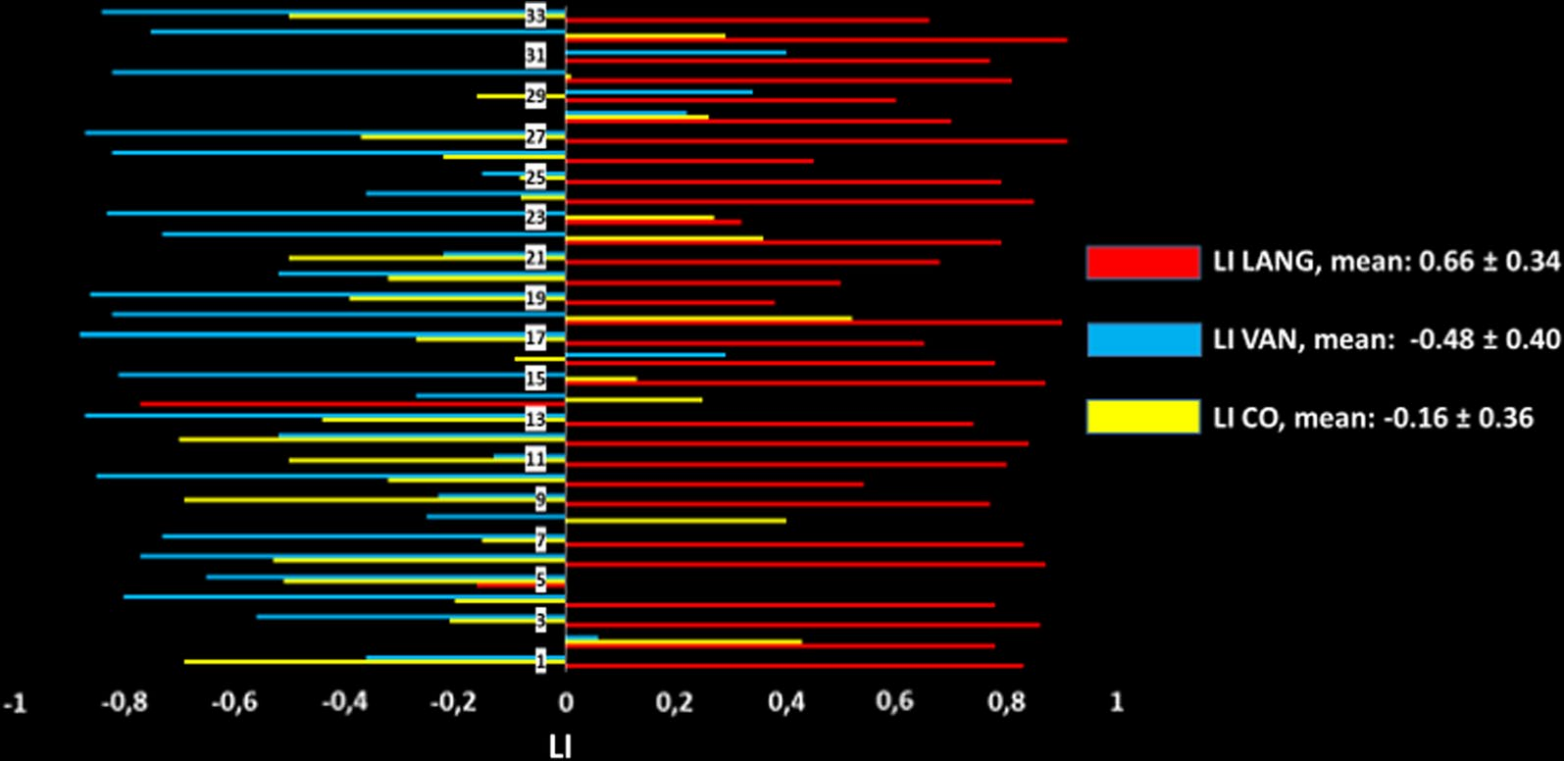
Sus-tentorial laterality index (LI) of the resting-state language (LANG), ventral attention (VAN) and cingulo-opercular (CO) networks identified by independent component analysis. Note the variability in the LI CO. The unique subject with a strong right lateralized LANG presents a lesser right lateralization of the VAN. This unique subject consistently shifted the LI leftward from the covert to overt session. Positive LI indicates left lateralization

At rest, the VAN and the CO were statistically right-lateralized (Fig. 5). The CO LI was only slightly right-lateralized with a mean value near zero (− 0.16) and thus, presented the greatest variability in the left–right categorization: 22 were right-lateralized and 10 were left-lateralized. The VAN was always more rightward lateralized than the LANG with the exception of one subject (subject 14) with the strongest right hemispheric lateralization of the LANG: the LI of this unique subject dramatically left-shifted from the Covert to Overt condition. This subject was non right-handed with an EIS of 0.20 [54] and had an LI of − 0.84 for the Covert contrast and thus fulfilled the criterion of strong atypical lateralization, i.e., inferior to − 0.50 [55]. Additional file 1: Table S2 shows all LI values, for each contrast and for each resting-state ICN, with the different used masks.

**Fig. 6:**
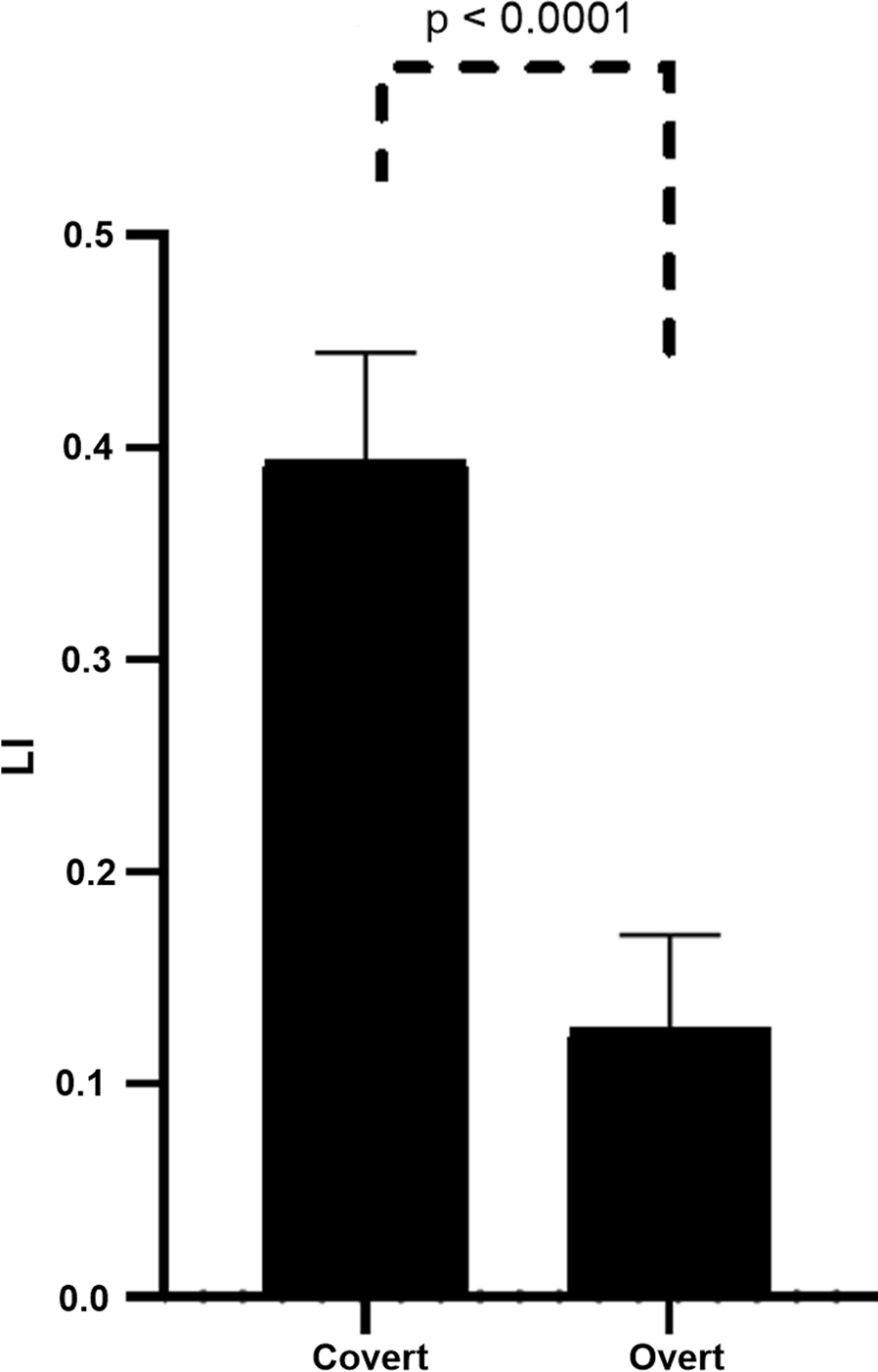
Two-sample t-test. Comparison of laterality index (LI) of the activity induced by the overt and covert linguistic tasks in the specific mask of sus-tentorial cingulo-opercular network as identified by spatial independent component analysis at rest

Additional file 1: Fig. S3 shows the pattern of activity for the Overt and Covert conditions in the specific mask of the three ICNs, i.e., the LANG, CO, and VAN. An overlap of language-induced activity was found within these ICNs in the right presupplementary motor area from y = 2 to y = 25. The activity induced by the linguistic task within the mask of the CO rightward shifted from the Covert to Overt condition (Fig. 6 and Additional file 1: Tables S3 and S4).

**Fig. 7:**
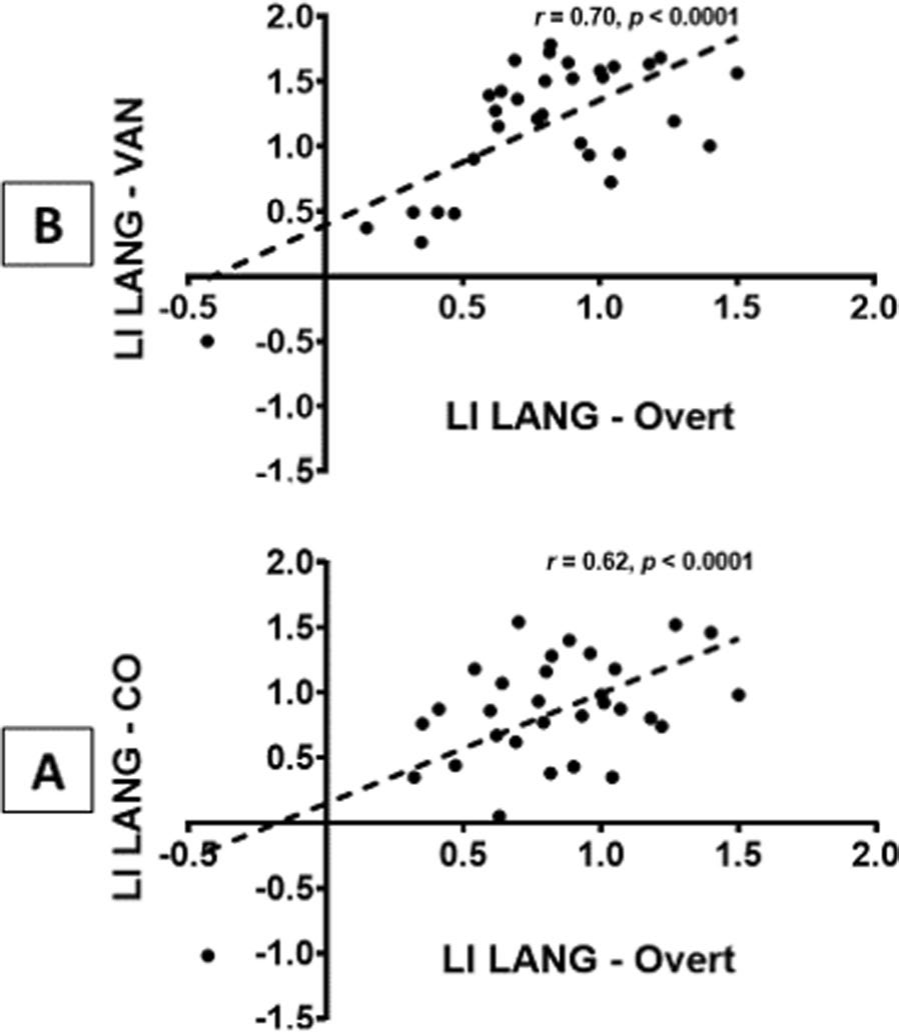
Linear correlations between the difference in the LI between the language network (LANG) as identified at rest by spatial independent component analysis and activity induced in the overt linguistic task (Overt) and the following: **A** the difference in the LI between the LANG and cingulo-opercular network (CO) identified at rest by spatial independent component analysis (n = 32). **B** the difference in the LI between the LANG and ventral attention network (VAN) identified at rest by spatial independent component analysis

A strong positive correlation was found between the difference in the LI LANG–Overt and the differences in the LI LANG–CO (*r* = 0.62, *p* < 0.0001) and LANG – VAN (*r* = 0.70, *p* < 0.0001). The greater the differences in lateralization at rest between the LANG and the CO or VAN, the greater the rightward shift in the language network when comparing the Overt condition to the LANG obtained by connectivity analysis (Fig. 7).

## Discussion

### Laterality index and overt speech

We found that the LI was significantly affected by the covert task compared to the resting-state LI of the language network but dramatically affected by the overt speech. This effect is so important that it is no longer possible to provide the basic information requested for clinical purpose on hemispheric lateralization of the language. Indeed, Fig. 4 shows that among our 29 volunteers classified as left-lateralized for the covert task, 18 were classified as right-lateralized for the overt task. Regarding the lateralization criteria of Mazoyer et al. [55], this generally results to classify as ambilateral, the typically left-lateralized subjects and the unique atypical subject.

We have previously shown [45] that the covert sentence generation task systematically coactivates the major nodes of the CO including the bilateral AIFO and the dorsal anterior cingulate. Such an activity pattern seems to be dependent on the difficult acoustic conditions inherent to MRI [56], but also to the “tonic” attentional load [57]. We identified the CO at rest together with the LANG and VAN. Among these three ICNs, the CO was slightly right-lateralized. Thus, systematic CO coactivation during the covert and overt speech contributed to the shift of the activity towards the right hemisphere.

Investigation of the LI in specific areas of interest in the frontal, parietal or temporal areas, while carefully excluding the pre and postcentral gyri, is of little help, as the same shifting pattern of activity from the left to right hemisphere was observed.

Such an effect suggests that the overt speaking (independently of the strong bilateral motor activity that tends to reduce the left lateralization of linguistic activity) recruits homologous areas in the right hemisphere. Indeed, this is the case as evidenced by Fig. 1, which shows the increased recruitment of the right superior temporal sulcus/superior temporal gyrus in the Overt condition. This appears to be related to the fact that prosodic pitch control involves the right hemisphere along ventral and dorsal pathways connected by white matter bundles similar to those found in the language network in the left hemisphere [38, 41].

### Attentional networks and lateralization

Performing spatial independent component analysis allowed us to isolate not only the language and cingulo-opercular networks but also a right-lateralized network presenting, in the opposite hemisphere, striking similarities with the language network. We referred to this network as the VAN [58, 59], involved in the detection of behaviorally relevant stimuli [60]. Interestingly, within this network, the pSTS appears to be activated by the pitch prosody [38]. FMRI studies have indicated that this area serves as an input region transferring prosodic acoustic information to frontal areas [61], but is also involved in facial action units recognition [62] and more generally activated by observed intentional actions [63]. Such a trans-modal activation of the right pSTS is reminiscent of its left counterpart in which speech is segmented into morphemic representations [64] and constitutes a main gate of entry into the language network for both visual and auditory inputs [15].

The major criterion used to discriminate at rest the atypical lateralized LANG from the VAN at the individual level was the analysis of activity within the inferior parietal cortex. This criterion appears robust as it allowed us to detect the LANG with high accuracy in patients eligible for awake surgery and electrical cortical mapping [45]. The present study further illustrated this finding, as shown in Fig. 2: a major anatomical difference was found in the inferior parietal cortex with the angular gyrus part of the LANG and the bilateral TPJ/SMG part of the VAN.

The sus-tentorial right hemispheric activity was present but less important in the Covert than in the Overt condition and was restricted in connectivity analysis to the temporal pole and the presupplementary motor area (preSMA). Together with a slight recruitment of the laryngeal phonologic areas during the covert speech (only identified at the group-level), this seems to indicate that even in the covert speech, there is an automatic representation and control of the speech prosody. Such activity could be due to the words’ presentation in our task. However, it has been shown that the pSTS is not only activated when listening words, but also when covertly producing and reading words, and play an important role in the speech through interaction with phonemics processing, subserved by the SMG in the left hemisphere [65].

Notably, in the 32 volunteers in whom we identified both the LANG and the VAN, 22 had opposite LI. Furthermore, in all except one subject, the VAN was always more right-lateralized than the LANG. In this unique subject, we also identified at rest a strong atypical right LANG lateralization and an atypical less right-shifted VAN than LANG. He also presented a major LI shift towards left during overt speech in accordance with the notion that, the regional pattern of rightward language asymmetries in Strong-atypicals is comparable and mirrors the regional pattern of leftward language asymmetries in Typicals [66].

Thus, the VAN lateralization at rest relative to the LANG plays a role in the shift in the LI observed in the overt speech condition. Indeed, we found a strong positive linear correlation between the difference in the LI LANG minus VAN at rest and the rightward shift in the LI in the Overt condition. It is noteworthy that this correlation was not due to an outlier, as it remained highly significant (*p* = 0.001) when excluding the unique subject with both LANG and VAN strong atypical lateralization.

Among the three networks we identified at rest, the CO is only slightly right-lateralized but presented the greatest variability across the subjects in the left–right classification. As in our precedent studies [12, 45], the CO appeared to be systematically coactivated during the covert and overt speech. A specificity of the CO is that it includes unique areas containing von Economo neurons within the dACC and AIFO. These neurons with long-range projections are found in the brain of great mammals [67, 68]. A proposed role for the CO is to segregate internal and extrapersonal stimuli to guide behavior, and a causal role of the CO has been found in switching from the default mode network to the central executive network [69, 70]. The CO BOLD signal has been found to positively correlate with electroencephalographic alpha-band power and appears to play an important role in attention through the control of inhibition [57, 71].

When carefully looking at the Fig. 1 and the Additional file 1: Tables S3 and S4, there appears to be a rightward shift of the activity from the Covert to Overt condition within the dACC, AIFO and putamen. As overt speaking involves the right hemispheric recruitment of the VAN, possible explanation for such a shift is the automatic increased activity of the right nodes of the CO inhibiting task irrelevant areas. Thus, similar to the VAN, the lateralization of the CO at rest equally appears to play a role in the rightward lateralization in the Overt condition, as indicated by the positive correlation found between the difference in the LI in LANG versus CO at rest and the shift in the LI in LANG versus Overt.

In summary, using the same ICA and visual expertise analysis that allowed validation of the LANG identification at rest, we were able to isolate two ICNs involved in cognitive control and attention. These ICNs are coactivated during explicit linguistic tasks, performed either covertly or overtly.

At this point, a question arises. How can these three ICNs coordinate their activity during explicit linguistic tasks? At rest, the three ICNs present a unique major overlap in the right caudal preSMA. The same overlap is found between the maps of the overt and covert tasks analyzed within the mask of the three ICNs, thus confirming that our linguistic task induces activity in an area common to the three ICNs. It has been shown that the preSMA is also involved in auditory processing. Specifically, the boundary between the preSMA and SMA appears to be involved in an effector-specific manner in the control of speech production (for a comprehensive review, see Lima et al. [72]). Our results seem to indicate a hemispheric asymmetry in the covert and overt treatment of the speech sounds properties, involving the right posterior preSMA. Indeed, Tables 1 and 2 indicate a major shift in the activity of the preSMA towards the right hemisphere during the overt sentence processing. In a previous study [46] on the connectivity of the preSMA within the language network, we found this area to be functionally connected to a bilateral and symmetric network overlapping the language network in the left hemisphere. The present study seems to confirm this finding and further suggests that the preSMA constitutes the space where the inherent conflicts between conditions and actions are resolved [73]. Thus, this process allows volitional control [74] by the coordinated activity among large scale networks. The mode of neuronal computation of conditions and actions within the preSMA is beyond the scope of the present study. However, biomathematical models confronted with in vivo data have indicated that the neurons of the medial prefrontal cortex present the property of mixed selectivity, characteristic of reservoir computing [75].

### Resting-state for laterality index?

We discussed the fact that the LI is strongly biased rightward during overt speech. It is noteworthy that in the covert speech, the LI is also moderately but significantly more rightward shifted compared to the LANG at rest. This leads to the opposite classification as left or right dominance in only two subjects. Such an agreement in 94% of our volunteers between the two imaging techniques seems reasonable compared to the 83.5% sensitivity and 88% specificity of the language-mapping task-fMRI against the Wada test [76]. However, it shall not be overlooked that there is no binary distribution of the LI, but more than two dominance categories involving ambilateral subjects [77]. Taking the cutoff of − 0.5 to + 0.18 to differentiate ambilateral subjects from lateralized subjects [55] leads to a disagreement in classification of more than half of our volunteers when comparing the LANG from rest to the covert task.

The feasibility of the isolation of the language network at rest by independent component analysis has been previously demonstrated [45, 46, 78–81] and validated against electrocortical mapping or the Wada test [45, 81, 82]. As we previously reported [45], we found that an important component of the language network, the left ANG, involved in semantic processing [15], is apparent at rest but not captured by an explicit task involving semantic processing. This is probably because, in the block design, semantic processing is present in both the task and non-interest periods of tones-listening, and semantic areas hardly achieve statistical significance when performing a two-sample t-test [45, 83]. This provides an argument for the superiority of connectivity analyses on block-designed tasks and contributes to the greater sensitivity of the former technique [45]. It is noteworthy that the LANG we isolated at rest by sICA shares strong similarities with the network of the 18-core sentences processing areas in an important recent paper [84] using the task-induced activity, graph analysis, and atlas of intrinsic connectivity of homotopic areas [85].

Furthermore, still in our recent paper, Lemée et al. [45], we found that the resting-state session had a sensitivity of 100% for the identification of eloquent brain language areas during intraoperative cortical mapping, whereas the sensitivity of task-based fMRI analysis was 65.6%. We could not isolate the language network at this high sensitivity in the resting-state session without the systematic identification of the VAN by sICA. By doing so, we found that the language network is distinct from the latter network handling the prosody [41].

Strong atypical language lateralization is a rare finding in a large cohort. Knecht et al. [86] estimated such occurrence in 27% in left-handers with a − 100 laterality on the handedness inventory score, which represent a minority among left-handers. Mazoyer et al. [55] reported this occurrence to be 7% in left-handers. The inclusion of an ambilateral group for language in 15% of the left-handers probably accounts for the difference with the study of Knecht et al.

Overall, it should be noted that in these studies, the language laterality was estimated by different technologies during the covert speech task (transcranial Doppler and fMRI) but are both based on the same physiological principles of hemodynamic changes.

The studies validating the transcranial Doppler against the Wada test should be taken cautiously due to the small cohorts and diverging results of meta-analysis, particularly in atypical patients [76, 87, 88].

In conclusion, we need to progress for clinical purpose in the determination of language lateralization in atypical patients, which constitute the target of functional investigation, and our data indicate that the coactivation of the CO and the VAN constitutes a bias in the calculation of the LI.

We propose that the LI be calculated on resting-state data by isolating the LANG by independent component analysis rather than on data obtained from explicit linguistic tasks. This also has the potential advantage of solving the problem of combining multiple specific tasks to cover several aspects of language at the risk of recruiting areas not essential to language.

Two previous studies challenged this issue in clinical patients with temporal lobe epilepsy [80, 81], however, only one confronted imaging data to the Wada test [81]. They found a sensitivity and specificity of 96% in a small cohort of 23 patients. It must be emphasized that these authors used a small dimensionality of components in their analysis and used an automated method to identify the LANG. They computed a Dice similarity index with a bilateral and symmetric template of language areas. Indeed, this methodology is exposed to the risk of selecting a single component aggregating the LANG and VAN. This is possibly at the origin of the misclassification they observed.

A future study comparing the LI of the LANG at rest with the Wada test in a large cohort should confirm the validity of this technique, particularly in patients with atypical lateralization. Such a study should also resolve whether recruitment of the nodes of the right-lateralized attentional ICNs is critical for language function.

### Limitations

Our study has some limitations. We proposed here to use the resting-state session instead of the task-based session in determining hemispheric dominance in neurosurgical patients. However, since this work only tested healthy volunteers, the ability of generalization to patients with brain tumor or epilepsy could be limited. Functional lateralization in patients may be different. In addition, motion artifacts may be greater in patients and can affect the results.

Furthermore, we cannot neglect the fact that there can be interindividual variability, as our main results are done at the group-level.

Finally, the study could be limited by the small number of participants.

## Conclusion

Although analyzing overt speech by fMRI may allow improvements in physiological knowledge on the coordinated activity of intrinsic connectivity networks for the achievement of the task, an overt speech task does not appear to be recommended for preoperative language mapping. The rightward shift of the LI in this condition did not provide the basic information on the hemispheric language dominance.

## Supplementary Information

**Additional file 1:** **Figure S1.** An example illustrating the paradigm used in our functional session. There were blocks of 16 seconds of word-sentences matching task (4 x 4 seconds) alternating with blocks of 16 seconds of tones-listening. A French random word (example: “Chien”) were audibly given to the subject who generated a short sentence (example: “Je promène mon chien”) semantically linked to the heard word. Each tone in the tones-listening block was a combination of three tones of increased frequency. **Figure S2.** Changes in the laterality index (LI) from Covert to Overt contrasts in specific brain areas. A: within the temporal mask. B: within the frontal mask excluding the precentral gyri. C: within the parietal mask excluding the postcentral gyri. Positive LI indicates left lateralization. **Figure S3.** Group-level one-sample t-test of the contrast covert speech production vs tone listening (A) and the contrast overt speech production vs tone listening (B), analyzed within the specific masks of the language (LANG), cingulo-opercular (CO) and ventral attention (VAN) networks. Images are projected on an MNI template. the left side of the brain is on the left. Images are shown corrected for multiple comparison FWE = 0.05 at the voxel-level with a minimum cluster extent k = 5. The color bar indicates T values. White numbers are the coordinates of the corresponding slices. In both the Covert and Overt contrasts, an intersection was found in a 208- and 389-voxel cluster, respectively, within right presupplementary motor area. **Table S1.** Maximal amplitudes of the 6 dimensions of head motion for the 33 subjects, in the overt and covert functional sessions. SD: standard deviation. **Table S2.** LI values for each contrast (Covert and Overt) and for each resting-state network (LANG, VAN, and CO), with and without the different used excluding masks. **Table S3.** One-sample t-test, group-level, 2nd order analysis of the contrast covert sentence generation vs tone listening within the mask FWE 0.05 corrected cluster-level (uncorrected 0.001 voxel-level) of the salience network as identified by functional connectivity analysis (n = 33). Peaks of activity are reported FWE 0.05 corrected at the voxel-level. **Table S4.** One-sample t-test, group-level, 2nd order analysis of the contrast overt sentence generation vs tone listening within the mask FWE 0.05 corrected cluster-level (uncorrected 0.001 voxel-level) of the salience network as identified by functional connectivity analysis (n = 33). Peaks of activity are reported FWE 0.05 corrected at the voxel-level.

## Data Availability

The datasets used and/or analyzed during the current study are available from the corresponding author on reasonable request.
