# Supplementary material for: Overt speech critically changes lateralization index and did not allow determination of hemispheric dominance for language: an fMRI study
Source: BMC Neurosci. 2021 Dec 1;22:74. doi: 10.1186/s12868-021-00671-y (PMC8638205; doi:10.1186/s12868-021-00671-y)
Supplement: Supplementary file 1 — Additional file 1: Figure S1. An example illustrating the paradigm used in our functional session. There were blocks of 16 seconds of word-sentences matching task (4 x 4 seconds) alternating with blocks of 16 seconds of tones-listening. A French random word (example: “Chien”) were audibly given to the subject who generated a short sentence (example: “Je promène mon chien”) semantically linked to the heard word. Each tone in the tones-listening block was a combination of three tones of increased frequency. Figure S2. Changes in the laterality index (LI) from Covert to Overt contrasts in specific brain areas. A: within the temporal mask. B: within the frontal mask excluding the precentral gyri. C: within the parietal mask excluding the postcentral gyri. Positive LI indicates left lateralization. Figure S3. Group-level one-sample t-test of the contrast covert speech production vs tone listening (A) and the contrast overt speech production vs tone listening (B), analyzed within the specific masks of the language (LANG), cingulo-opercular (CO) and ventral attention (VAN) networks. Images are projected on an MNI template. the left side of the brain is on the left. Images are shown corrected for multiple comparison FWE = 0.05 at the voxel-level with a minimum cluster extent k = 5. The color bar indicates T values. White numbers are the coordinates of the corresponding slices. In both the Covert and Overt contrasts, an intersection was found in a 208- and 389-voxel cluster, respectively, within right presupplementary motor area. Table S1. Maximal amplitudes of the 6 dimensions of head motion for the 33 subjects, in the overt and covert functional sessions. SD: standard deviation. Table S2. LI values for each contrast (Covert and Overt) and for each resting-state network (LANG, VAN, and CO), with and without the different used excluding masks. Table S3. One-sample t-test, group-level, 2nd order analysis of the contrast covert sentence generation vs tone listening within the [file 12868_2021_671_MOESM1_ESM.docx]

Supplementary Material

Overt speech unravels cooperation between language and attentional networks and critically changes lateralization index: an fMRI study

David Hassanein Berro, Jean-Michel Lemée, Louis-Marie Leiber, Evelyne Emery, Philippe Menei, Aram Ter Minassian


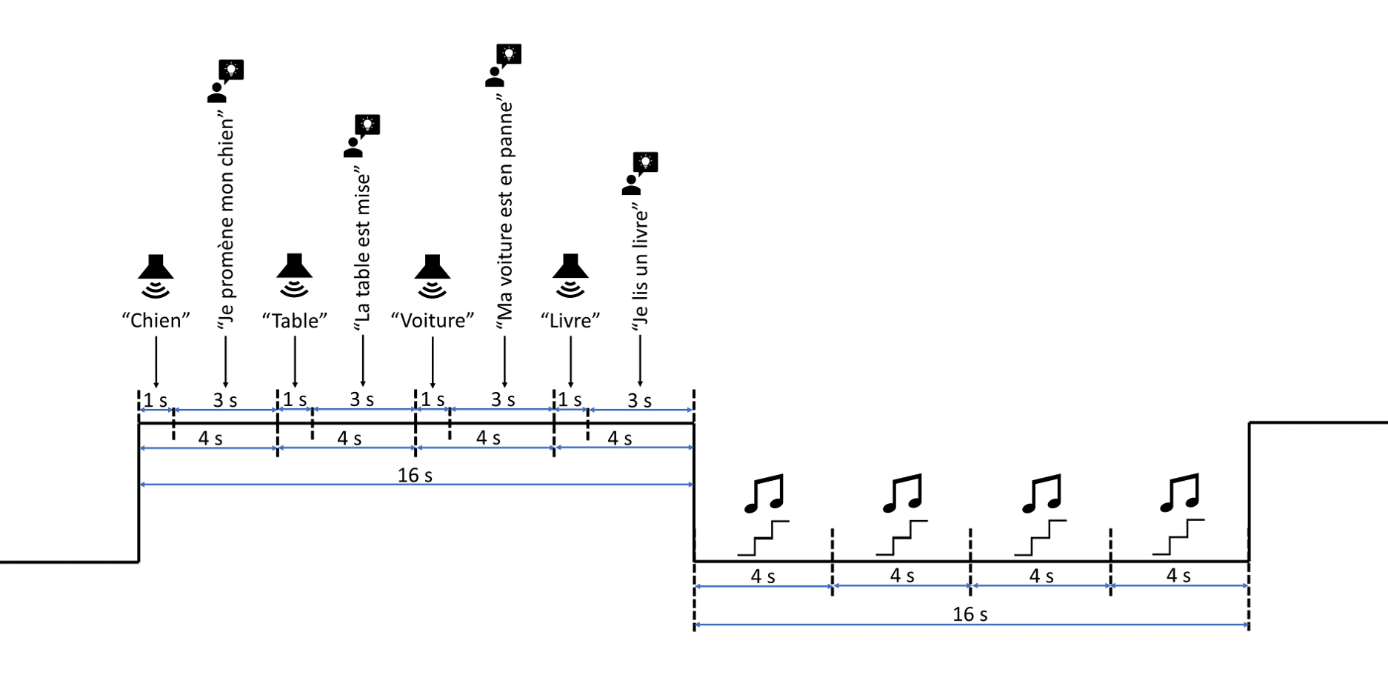
 **Fig. S1** An example illustrating the paradigm used in our functional session. There were blocks of 16 seconds of word-sentences matching task (4 x 4 seconds) alternating with blocks of 16 seconds of tones-listening. A French random word (example: “Chien”) were audibly given to the subject who generated a short sentence (example: “Je promène mon chien”) semantically linked to the heard word. Each tone in the tones-listening block was a combination of three tones of increased frequency. Translation of French words:

- “Chien”: Dog; “Je promène mon chien”: I take my dog for a walk
- “Table”: Table; “La table est mise”: The table is set
- “Voiture”: Car; “Ma voiture est en panne”: My car is broken down
- “Livre”: Book; “Je lis un livre”: I’m reading a book.

This figure was originally published in our previous paper: “Berro DH, Lemée JM, Leiber LM, Emery E, Menei P, Ter Minassian A. Overt speech feasibility using continuous functional magnetic resonance imaging: Isolation of areas involved in phonology and prosody. J Neurosci Res. 2020;98:2554–65”.


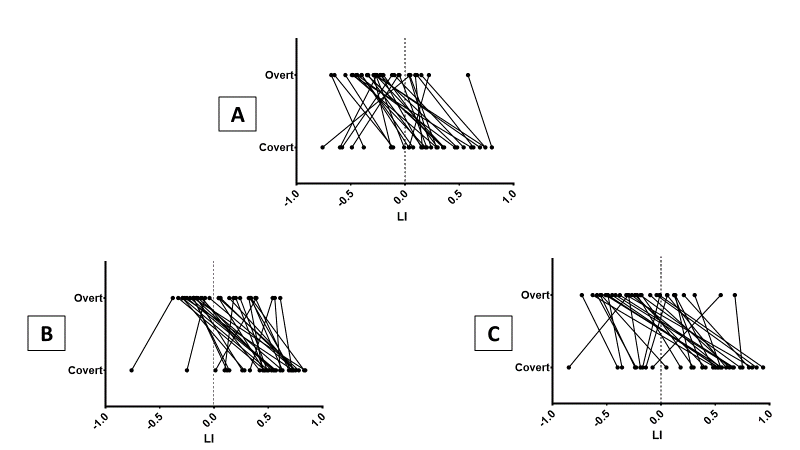


Fig. S2 Changes in the laterality index (LI) from Covert to Overt contrasts in specific brain areas. A: within the temporal mask. B: within the frontal mask excluding the precentral gyri. C: within the parietal mask excluding the postcentral gyri. Positive LI indicates left lateralization.


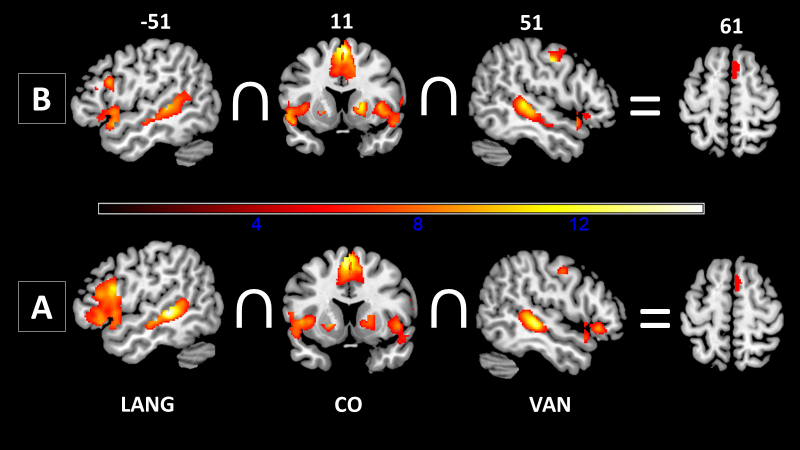


Fig. S3 Group-level one-sample t-test of the contrast covert speech production vs tone listening (A) and the contrast overt speech production vs tone listening (B), analyzed within the specific masks of the language (LANG), cingulo-opercular (CO) and ventral attention (VAN) networks. Images are projected on an MNI template. the left side of the brain is on the left. Images are shown corrected for multiple comparison FWE = 0.05 at the voxel-level with a minimum cluster extent k = 5. The color bar indicates T values. White numbers are the coordinates of the corresponding slices. In both the Covert and Overt contrasts, an intersection was found in a 208- and 389-voxel cluster, respectively, within right presupplementary motor area.

Table S1 Maximal amplitudes of the 6 dimensions of head motion for the 33 subjects, in the overt and covert functional sessions. SD: standard deviation.

| **Overt Session** | | | | | |  | **Covert Session** | | | | | |
| --- | --- | --- | --- | --- | --- | --- | --- | --- | --- | --- | --- | --- |
| **x** | **y** | **z** | **pitch** | **roll** | **yaw** | **Subject** | **x** | **y** | **z** | **pitch** | **roll** | **yaw** |
| 0.168 | 0.833 | 0.683 | 0.007 | 0 | 0.002 | **1** | 0.243 | 0.142 | 0.693 | 0.001 | 0.001 | 0.004 |
| 0.003 | 0.057 | 1.013 | 0.015 | 0 | 0.001 | **2** | 0.081 | 0.045 | 0.206 | 0.002 | 0.001 | 0.003 |
| 0.168 | 0.316 | 2.269 | 0.01 | 0.006 | 0.008 | **3** | 0.149 | 0.051 | 0.723 | 0.002 | 0.001 | 0 |
| 0.028 | 0.055 | 0.281 | 0.009 | 0.002 | 0.001 | **4** | 0.023 | 0.388 | 0.458 | 0.018 | 0.003 | 0.001 |
| 0.031 | 0.078 | 0.806 | 0.009 | 0.004 | 0.001 | **5** | 0.025 | 0.001 | 0.388 | 0.005 | 0.001 | 0.001 |
| 0.004 | 0.012 | 0.329 | 0.003 | 0.005 | 0 | **6** | 0.016 | 0.077 | 0.35 | 0.002 | 0.004 | 0.004 |
| 0.031 | 0.345 | 0.366 | 0.018 | 0.001 | 0.003 | **7** | 0.02 | 0.022 | 0.201 | 0.001 | 0.003 | 0.001 |
| 0.195 | 0.092 | 0.19 | 0 | 0.001 | 0.004 | **8** | 0.016 | 0.06 | 0.173 | 0.002 | 0.002 | 0.001 |
| 0.015 | 0.296 | 1.911 | 0.005 | 0.003 | 0.001 | **9** | 0.101 | 0.464 | 0.721 | 0.008 | 0.001 | 0.001 |
| 0.818 | 0.153 | 1.032 | 0 | 0.025 | 0.009 | **10** | 0.132 | 0.008 | 0.332 | 0.003 | 0.001 | 0 |
| 0.217 | 0.66 | 0.575 | 0.017 | 0.002 | 0.006 | **11** | 0.172 | 0.089 | 0.274 | 0 | 0.002 | 0.004 |
| 0.066 | 0.398 | 0.218 | 0.008 | 0.007 | 0.003 | **12** | 0.302 | 0.381 | 0.187 | 0.007 | 0.001 | 0.006 |
| 0.182 | 0.053 | 0.451 | 0.007 | 0.004 | 0.001 | **13** | 0.023 | 0.163 | 0.118 | 0.005 | 0.001 | 0 |
| 0.12 | 0.566 | 2.049 | 0.013 | 0.002 | 0.001 | **14** | 0.044 | 0.249 | 0.174 | 0.007 | 0.002 | 0.003 |
| 0.036 | 0.023 | 0.621 | 0.002 | 0.004 | 0 | **15** | 0.043 | 0.116 | 0.02 | 0.005 | 0.001 | 0.003 |
| 0.048 | 0.201 | 0.132 | 0.007 | 0.002 | 0 | **16** | 0.11 | 0.057 | 0.994 | 0.017 | 0.002 | 0.003 |
| 0.055 | 0.073 | 0.096 | 0.001 | 0.001 | 0 | **17** | 0.018 | 0.008 | 0.048 | 0.001 | 0 | 0 |
| 0.213 | 0.87 | 1.246 | 0.022 | 0.027 | 0.01 | **18** | 0.295 | 0.046 | 0.881 | 0.009 | 0.008 | 0.005 |
| 0.165 | 0.015 | 0.083 | 0.001 | 0.007 | 0.002 | **19** | 0.066 | 0.065 | 0.172 | 0.006 | 0 | 0.001 |
| 0.1 | 0.317 | 0.224 | 0.011 | 0.002 | 0.004 | **20** | 0.066 | 0.368 | 0.285 | 0.003 | 0.005 | 0 |
| 0.098 | 0.046 | 0.162 | 0.003 | 0.005 | 0.001 | **21** | 0.27 | 0.022 | 0.247 | 0.004 | 0.001 | 0.002 |
| 0.067 | 0.057 | 0.006 | 0.002 | 0.003 | 0.001 | **22** | 0.151 | 0.041 | 0.054 | 0.002 | 0.004 | 0.002 |
| 0.076 | 0.24 | 0.269 | 0 | 0.002 | 0.004 | **23** | 0.055 | 0.101 | 0.322 | 0.005 | 0.001 | 0.001 |
| 0.092 | 0.874 | 0.989 | 0.009 | 0.001 | 0.005 | **24** | 0.004 | 0.08 | 0.06 | 0.003 | 0.001 | 0.006 |
| 0.068 | 0.047 | 0 | 0.005 | 0.001 | 0 | **25** | 0.056 | 0.054 | 0.349 | 0.012 | 0.01 | 0 |
| 0.042 | 0.449 | 0 | 0.001 | 0.006 | 0.003 | **26** | 0.208 | 0.131 | 0.031 | 0.001 | 0.001 | 0.001 |
| 0.026 | 0.108 | 0.099 | 0.001 | 0 | 0.001 | **27** | 0.093 | 0.11 | 0.1 | 0.003 | 0.002 | 0.002 |
| 0.125 | 0.118 | 0.187 | 0.013 | 0.002 | 0.004 | **28** | 0.029 | 0.31 | 0.882 | 0.012 | 0.001 | 0.002 |
| 0.061 | 0.118 | 0.006 | 0.005 | 0.001 | 0.001 | **29** | 0.172 | 0.017 | 0.159 | 0.001 | 0.002 | 0 |
| 0.065 | 0.414 | 0.785 | 0.017 | 0.001 | 0.002 | **30** | 0.093 | 0.07 | 0.159 | 0.003 | 0.005 | 0.001 |
| 0.127 | 0.112 | 0.568 | 0.003 | 0.003 | 0.004 | **31** | 0.082 | 0.037 | 0.095 | 0.001 | 0.003 | 0.002 |
| 0.195 | 0.206 | 0.519 | 0.012 | 0.008 | 0.005 | **32** | 0.192 | 0.267 | 0.692 | 0.008 | 0.006 | 0.007 |
| 0.066 | 0.356 | 0.011 | 0.008 | 0.002 | 0.001 | **33** | 0.078 | 0.039 | 0.33 | 0.008 | 0.002 | 0.001 |
|  |  |  |  |  |  |  |  |  |  |  |  |  |
| 0.114 | 0.259 | 0.551 | 0.008 | 0.004 | 0.003 | **Mean** | 0.104 | 0.124 | 0.33 | 0.005 | 0.002 | 0.002 |
| 0.142 | 0.254 | 0.599 | 0.006 | 0.006 | 0.003 | **SD** | 0.086 | 0.128 | 0.274 | 0.005 | 0.002 | 0.002 |

This table was originally published in the supplementary material of our previous paper: “Berro DH, Lemée JM, Leiber LM, Emery E, Menei P, Ter Minassian A. Overt speech feasibility using continuous functional magnetic resonance imaging: Isolation of areas involved in phonology and prosody. J Neurosci Res. 2020;98:2554–65”.

Table S2 LI values for each contrast (Covert and Overt) and for each resting-state network (LANG, VAN, and CO), with and without the different used excluding masks.

|  | **Covert** | | | | | | **Overt** | | | | | | **LANG** | | **CO** | | **VAN** | |
| --- | --- | --- | --- | --- | --- | --- | --- | --- | --- | --- | --- | --- | --- | --- | --- | --- | --- | --- |
| **Subject** | **Whole brain** | **Sus-tentorial** | **Frontal** | **Frontal,**  **preCG excluded** | **Parietal** | **Parietal,**  **postCG excluded** | **Whole brain** | **Sus-tentorial** | **Frontal** | **Frontal,**  **preCG excluded** | **Parietal** | **Parietal,**  **postCG excluded** | **Whole brain** | **Sus-tentorial** | **Whole brain** | **Sus-tentorial** | **Whole brain** | **Sus-tentorial** |
| **1** | 0.051 | 0.031 | 0.13 | 0.47 | -0.19 | -0.24 | -0.38 | -0.44 | -0.46 | -0.33 | -0.59 | -0.56 | 0.78 | 0.83 | -0.68 | -0.69 | -0.37 | -0.36 |
| **2** | 0.35 | 0.85 | 0.88 | 0.84 | 0.58 | 0.5 | -0.32 | -0.26 | -0.31 | -0.19 | -0.37 | -0.63 | 0.69 | 0.78 | 0.43 | 0.43 | 0.094 | 0.059 |
| **3** | 0.035 | 0.36 | 0.49 | 0.45 | 0.24 | 0.28 | 0.22 | 0.22 | 0.3 | 0.32 | 0.21 | 0.13 | 0.85 | 0.86 | -0.21 | -0.21 | -0.54 | -0.56 |
| **4** | -0.012 | 0.012 | -0.013 | 0.26 | 0.14 | 0.38 | -0.25 | -0.22 | -0.31 | -0.16 | -0.22 | 0.12 | 0.74 | 0.78 | -0.2 | -0.2 | -0.75 | -0.8 |
| **5** | -0.18 | -0.28 | -0.27 | -0.25 | -0.091 | -0.23 | -0.43 | -0.48 | -0.35 | -0.083 | -0.19 | -0.59 | -0.17 | -0.16 | 0.69 | -0.51 | -0.65 | -0.65 |
| **6** | 0.56 | 0.78 | 0.81 | 0.78 | 0.94 | 0.94 | -0.075 | -0.014 | 0.012 | 0.044 | 0.017 | -0.016 | 0.85 | 0.87 | -0.52 | -0.53 | -0.76 | -0.77 |
| **7** | -0.41 | -0.41 | 0.12 | 0.53 | 0.66 | 0.66 | -0.63 | -0.67 | -0.45 | -0.12 | -0.44 | -0.42 | 0.75 | 0.83 | -0.15 | -0.15 | -0.71 | -0.73 |
| **8** | 0.55 | 0.77 | 0.78 | 0.73 | 0.57 | 0.53 | -0.43 | -0.42 | -0.42 | -0.27 | -0.49 | -0.49 |  |  | 0.39 | 0.4 | -0.75 | -0.76 |
| **9** | 0.018 | 0.037 | 0.56 | 0.56 | -0.14 | 0.049 | -0.61 | -0.63 | -0.44 | -0.25 | -0.3 | -0.55 | 0.76 | 0.77 | -0.68 | -0.69 | -0.22 | -0.23 |
| **10** | 0.039 | 0.46 | 0.77 | 0.75 | 0.77 | 0.84 | -0.35 | -0.057 | 0.049 | 0.06 | -0.32 | -0.32 | 0.47 | 0.54 | -0.32 | -0.32 | -0.79 | -0.85 |
| **11** | 0.49 | 0.56 | 0.63 | 0.75 | 0.68 | 0.56 | -0.17 | -0.16 | -0.2 | 0.14 | -0.18 | -0.51 | 0.68 | 0.8 | -0.49 | -0.5 | -0.13 | -0.13 |
| **12** | 0.2 | 0.3 | 0.49 | 0.49 | -0.14 | -0.14 | 0.042 | 0.14 | 0.32 | 0.24 | 0.48 | 0.058 | 0.76 | 0.84 | -0.69 | -0.7 | -0.52 | -0.52 |
| **13** | 0.51 | 0.53 | 0.47 | 0.42 | 0.75 | 0.67 | -0.34 | -0.31 | -0.29 | -0.11 | -0.25 | -0.38 | 0.66 | 0.74 | -0.44 | -0.44 | -0.8 | -0.87 |
| **14** | -0.65 | -0.84 | -0.79 | -0.76 | -0.8 | -0.85 | -0.26 | -0.34 | -0.5 | -0.38 | 0.33 | -0.3 | -0.74 | -0.77 | 0.23 | 0.25 | -0.27 | -0.27 |
| **15** | 0.41 | 0.46 | 0.56 | 0.57 | 0.47 | 0.63 | -0.47 | -0.35 | -0.34 | -0.22 | -0.079 | -0.1 | 0.85 | 0.87 | 0.11 | 0.13 | -0.8 | -0.81 |
| **16** | 0.39 | 0.4 | 0.59 | 0.61 | 0.26 | -0.17 | 0.37 | 0.37 | 0.49 | 0.56 | 0.29 | -0.011 | 0.73 | 0.78 | -0.092 | -0.092 | 0.28 | 0.29 |
| **17** | 0.4 | 0.43 | 0.53 | 0.63 | 0.47 | -0.19 | -0.35 | -0.36 | -0.34 | -0.15 | -0.18 | -0.3 | 0.43 | 0.65 | -0.27 | -0.27 | -0.85 | -0.88 |
| **18** | -0.13 | 0.71 | 0.79 | 0.83 | 0.52 | 0.41 | -0.098 | 0.084 | 0.27 | 0.34 | 0.087 | -0.24 | 0.63 | 0.9 | 0.44 | 0.52 | -0.77 | -0.82 |
| **19** | -0.11 | -0.094 | 0.34 | 0.12 | -0.34 | -0.36 | -0.43 | -0.41 | -0.21 | -0.19 | -0.29 | -0.49 | 0.39 | 0.38 | -0.38 | -0.39 | -0.85 | -0.86 |
| **20** | 0.3 | 0.31 | 0.12 | 0.28 | 0.52 | 0.52 | -0.44 | -0.43 | -0.39 | -0.29 | -0.37 | -0.45 | 0.48 | 0.5 | -0.32 | -0.32 | -0.52 | -0.52 |
| **21** | 0.51 | 0.69 | 0.67 | 0.69 | 0.64 | 0.6 | 0.14 | 0.14 | 0.1 | 0.38 | 0.37 | 0.055 | 0.63 | 0.68 | -0.49 | -0.5 | -0.21 | -0.22 |
| **22** | 0.11 | 0.26 | 0.33 | 0.46 | 0.4 | 0.55 | -0.4 | -0.11 | -0.096 | 0.043 | -0.54 | -0.27 | 0.78 | 0.79 | 0.36 | 0.36 | -0.72 | -0.73 |
| **23** | 0.086 | 0.098 | 0.15 | 0.098 | 0.35 | 0.56 | -0.29 | -0.31 | 0.046 | 0.18 | 0.054 | -0.5 | 0.29 | 0.32 | 0.27 | 0.27 | -0.79 | -0.83 |
| **24** | 0.64 | 0.66 | 0.74 | 0.71 | 0.88 | 0.88 | 0.056 | 0.078 | 0.17 | 0.33 | 0.15 | -0.041 | 0.83 | 0.85 | -0.081 | -0.081 | -0.36 | -0.36 |
| **25** | 0.31 | 0.31 | -0.044 | 0.14 | 0.5 | 0.18 | -0.28 | -0.28 | -0.18 | -0.19 | -0.15 | -0.22 | 0.76 | 0.79 | -0.08 | -0.083 | -0.15 | -0.15 |
| **26** | 0.45 | 0.64 | 0.81 | 0.75 | 0.78 | 0.81 | -0.25 | -0.17 | -0.13 | -0.042 | 0.02 | 0.21 | 0.4 | 0.45 | -0.22 | -0.22 | -0.74 | -0.82 |
| **27** | 0.31 | 0.28 | 0.54 | 0.55 | 0.33 | 0.3 | -0.078 | 0.09 | 0.12 | -0.18 | 0.21 | -0.18 | 0.83 | 0.91 | -0.37 | -0.37 | -0.86 | -0.87 |
| **28** | 0.67 | 0.68 | 0.67 | 0.63 | 0.69 | 0.73 | 0.2 | 0.23 | 0.27 | 0.34 | 0.42 | -0.19 | 0.69 | 0.7 | 0.26 | 0.26 | 0.23 | 0.22 |
| **29** | 0.1 | 0.098 | 0.034 | 0.013 | 0.51 | 0.48 | 0.23 | 0.25 | 0.26 | 0.39 | 0.36 | 0.13 | 0.55 | 0.6 | -0.16 | -0.16 | 0.34 | 0.34 |
| **30** | 0.36 | 0.43 | 0.57 | 0.5 | -0.37 | -0.4 | -0.37 | -0.37 | -0.43 | -0.29 | -0.57 | -0.73 | 0.8 | 0.81 | 0.011 | 0.0098 | -0.8 | -0.82 |
| **31** | 0.76 | 0.86 | 0.74 | 0.72 | 0.75 | 0.75 | 0.44 | 0.62 | 0.53 | 0.61 | 0.75 | 0.68 | 0.51 | 0.77 |  |  | 0.41 | 0.4 |
| **32** | -0.19 | 0.13 | 0.42 | 0.33 | 0.28 | -0.078 | -0.0098 | 0.22 | 0.73 | 0.54 | 0.67 | 0.55 | 0.73 | 0.91 | 0.31 | 0.29 | -0.72 | -0.75 |
| **33** | 0.21 | 0.35 | 0.4 | 0.46 | 0.56 | 0.55 | -0.11 | -0.14 | 0.2 | 0.2 | 0.27 | 0.31 | 0.59 | 0.66 | -0.43 | -0.5 | -0.82 | -0.84 |

CO: cingulo-opercular network, LANG: language network, postCG: postcentral gyrus, preCG: precentral gyrus, VAN: ventral attention network.

Table S3 One-sample t-test, group-level, 2^nd^ order analysis of the contrast covert sentence generation vs tone listening within the mask FWE 0.05 corrected cluster-level (uncorrected 0.001 voxel-level) of the salience network as identified by functional connectivity analysis (n = 33). Peaks of activity are reported FWE 0.05 corrected at the voxel-level.

|  | **k** | **t_32_** | **x** | **y** | **z** |
| --- | --- | --- | --- | --- | --- |
| **L preSMA** | **4149** | 15.27 | -3 | 6 | 56 |
| **L preSMA** |  | 14.99 | -3 | 5 | 65 |
| **L preSMA** |  | 10.79 | -5 | 14 | 48 |
| **L MCC/dACC** |  | 10.27 | -8 | 18 | 38 |
| **L preSMA** |  | 9.82 | -9 | 6 | 68 |
| **R preSMA** |  | 9.73 | 9 | 12 | 53 |
| **R MCC/dACC** |  | 8.28 | 9 | 20 | 33 |
| **R ACC** |  | 6.64 | 9 | 20 | 24 |
| **L Insula Lobe (AIFO)** | **2934** | 11.28 | -42 | 17 | 0 |
| **L Insula Lobe (AIFO)** |  | 10.73 | -29 | 21 | 2 |
| **L Putamen** |  | 9.92 | -18 | 9 | -2 |
| **L IFG (p. Opercularis)** |  | 9.82 | -47 | 11 | 2 |
| **L Insula Lobe** |  | 9.07 | -47 | 6 | 3 |
| **L IFG (p. Orbitalis)** |  | 9.05 | -47 | 20 | -11 |
| **L IFG (p. Orbitalis)** |  | 9.01 | -50 | 24 | -8 |
| **L Pallidum** |  | 8.76 | -14 | 9 | 3 |
| **L IFG (p. Opercularis)** |  | 8.25 | -54 | 14 | 11 |
| **L IFG (p. Opercularis)** |  | 8.21 | -54 | 9 | 15 |
| **R Insula Lobe** | **1760** | 8.99 | 47 | 11 | -3 |
| **R Insula Lobe (AIFO)** |  | 8.23 | 39 | 17 | 2 |
| **R IFG (p. Orbitalis)** |  | 8.2 | 51 | 24 | -6 |
| **R Temporal Pole** |  | 7.75 | 47 | 17 | -15 |
| **R Insula Lobe (AIFO)** |  | 7.55 | 33 | 18 | 5 |
| **R Temporal Pole** |  | 7.08 | 59 | 8 | -12 |
| **R IFG (p. Orbitalis)** |  | 7.06 | 35 | 24 | -12 |
| **R IFG (p. Orbitalis)** |  | 6.92 | 47 | 29 | -3 |
| **R Caudate Nucleus** | **380** | 10.63 | 18 | 15 | 8 |
| **R Pallidum** |  | 7.58 | 14 | 6 | -2 |
| **R Cerebellum (VI)** |  | 7.95 | 29 | -62 | -24 |
| **R Cerebellum (VI)** |  | 7.68 | 29 | -69 | -26 |
| **R Cerebellum (VI)** |  | 6.47 | 20 | -66 | -20 |
| **L Cerebellum (VI)** | **211** | 8.32 | -30 | -59 | -29 |
| **L Cerebellum (Crus 1)** |  | 7.55 | -41 | -59 | -33 |
| **L Cerebellum (VI)** |  | 7.05 | -29 | -56 | -33 |
| **L Cerebellum (VI)** |  | -23 | -23 | -60 | -24 |
| **R Cerebellum (Crus I)** | **187** | 9.91 | 35 | -57 | -30 |
| **L Middle Frontal Gyrus** | **113** | 6.92 | -30 | 47 | 18 |
| **R Middle Frontal Gyrus** | **46** | 6.89 | 33 | 42 | 18 |
| **L Thalamus** | **37** | 7.69 | -9 | -17 | 8 |
| **R Superior Temporal Gyrus** | **21** | 7.42 | 60 | 0 | -8 |
| **R IFG (p. Opercularis)** | **15** | 6.27 | 59 | 14 | 23 |
| **R IFG (p. Opercularis)** |  | 5.92 | 59 | 8 | 23 |
| **R Rolandic Operculum** | **10** | 6.14 | 62 | 8 | 14 |
| **L Precentral Gyrus** | **6** | 5.8 | -59 | 6 | 23 |

k: cluster extent in voxel number. t_32_: t value for 32 degrees of freedom. x, y, z: coordinates in MNI space. ACC: anterior cingulate cortex, AIFO: anterior insula frontal operculum, IFG: inferior frontal gyrus, MCC: middle cingulate cortex, SMA: supplementary motor area.

Table S4 One-sample t-test, group-level, 2^nd^ order analysis of the contrast overt sentence generation vs tone listening within the mask FWE 0.05 corrected cluster-level (uncorrected 0.001 voxel-level) of the salience network as identified by functional connectivity analysis (n = 33). Peaks of activity are reported FWE 0.05 corrected at the voxel-level.

|  | **k** | **t_32_** | **x** | **y** | **z** |
| --- | --- | --- | --- | --- | --- |
| **R preSMA** | **5329** | 15.33 | 2 | 8 | 63 |
| **L MCC/dACC** |  | 12.84 | -8 | 20 | 33 |
| **R MCC/dACC** |  | 10.9 | 12 | 17 | 32 |
| **R MCC** |  | 10.2 | 11 | 15 | 44 |
| **L MCC** |  | 10.07 | -5 | 15 | 41 |
| **L preSMA** |  | 9.56 | -6 | 6 | 72 |
| **R preSMA** |  | 9.52 | 3 | 21 | 56 |
| **R preSMA** |  | 9.42 | 9 | 12 | 51 |
| **L ACC** |  | 5.99 | -11 | 30 | 23 |
| **R preSMA** |  | 5.94 | 11 | 6 | 71 |
| **L Insula Lobe (AIFO)** | **2785** | 10.43 | -29 | 21 | 2 |
| **L Temporal Pole** |  | 10.06 | -56 | 9 | -8 |
| **L Temporal Pole** |  | 9.82 | -50 | 11 | -12 |
| **L IFG (p. Orbitalis)** |  | 9.19 | -44 | 21 | -11 |
| **L Temporal Pole** |  | 9.19 | -53 | 11 | -2 |
| **L Insula Lobe** |  | 9.09 | -38 | 21 | -8 |
| **L IFG (p. Orbitalis)** |  | 9.06 | -39 | 20 | -6 |
| **L Insula Lobe (AIFO)** |  | 8.83 | -26 | 20 | 6 |
| **L Temporal Pole** |  | 8.57 | -48 | 17 | -12 |
| **L Superior Temporal Gyrus** |  | 8.4 | -59 | 3 | -5 |
| **L Superior Temporal Gyrus** |  | 8.27 | -60 | -12 | 5 |
| **R Putamen** | **2777** | 12.33 | 20 | 12 | 3 |
| **R Superior Temporal Gyrus** |  | 11.75 | 62 | -5 | -2 |
| **R Insula Lobe (AIFO)** |  | 11.12 | 39 | 14 | 5 |
| **R Temporal Pole** |  | 9.79 | 51 | 14 | -14 |
| **R Insula Lobe** |  | 9.01 | 44 | 11 | -3 |
| **R Precentral Gyrus** |  | 8.91 | 59 | 3 | 20 |
| **R Insula Lobe (AIFO)** |  | 8.9 | 33 | 18 | 5 |
| **R Rolandic Operculum** |  | 8.21 | 59 | 0 | 11 |
| **R Temporal Pole** |  | 7.8 | 59 | 8 | -9 |
| **R Cerebellum (VI)** | **415** | 10.86 | 20 | -72 | -24 |
| **R Cerebellum (VI)** |  | 10.4 | 18 | -68 | -18 |
| **R Cerebellum (VI)** |  | 10.03 | 23 | -65 | -21 |
| **R Cerebellum (VI)** |  | 9.81 | 29 | -62 | -24 |
| **R Cerebellum (VI)** |  | 9.21 | 32 | -69 | -24 |
| **L Cerebellum (VI)** | **381** | 10.35 | -21 | -57 | -24 |
| **L Cerebellum (VI)** |  | 10.34 | -32 | -59 | -29 |
| **L Cerebellum (VI)** |  | 10.19 | -24 | -62 | -23 |
| **L Cerebellum (Crus 1)** |  | 6.38 | -41 | -59 | -33 |
| **L Cerebellum (VI)** |  | 6.25 | -32 | -56 | -33 |
| **L Middle Frontal Gyrus** | **259** | 7.21 | -29 | 45 | 21 |
| **L Middle Frontal Gyrus** |  | 6.25 | -24 | 41 | 18 |
| **L Middle Frontal Gyrus** |  | 5.76 | -29 | 45 | 9 |
| **R Cerebellum (VI)** | **124** | 9.14 | 33 | -57 | -29 |
| **R Cerebellum (Crus 1)** |  | 9.03 | 38 | -57 | -30 |
| **L Putamen** | **107** | 8.87 | -20 | 8 | -2 |
| **L Pallidum** |  | 6.46 | -14 | 9 | 3 |
| **L Postcentral Gyrus** | **73** | 9.61 | -59 | 0 | 20 |
| **R Middle Frontal Gyrus** | **53** | 6.54 | 32 | 41 | 18 |
| **L Thalamus** | **8** | 5.77 | -11 | -18 | 8 |

k: cluster extent in voxel number. t_32_: t value for 32 degrees of freedom. x, y, z: coordinates in MNI space. ACC: anterior cingulate cortex, AIFO: anterior insula frontal operculum, IFG: inferior frontal gyrus, MCC: middle cingulate cortex, SMA: supplementary motor area.
